# Ensembles of Spiking Neurons with Noise Support Optimal Probabilistic Inference in a Dynamically Changing Environment

**DOI:** 10.1371/journal.pcbi.1003859

**Published:** 2014-10-23

**Authors:** Robert Legenstein, Wolfgang Maass

**Affiliations:** Institute for Theoretical Computer Science, Graz University of Technology, Graz, Austria; Indiana University, United States of America

## Abstract

It has recently been shown that networks of spiking neurons with noise can emulate simple forms of probabilistic inference through “neural sampling”, i.e., by treating spikes as samples from a probability distribution of network states that is encoded in the network. Deficiencies of the existing model are its reliance on single neurons for sampling from each random variable, and the resulting limitation in representing quickly varying probabilistic information. We show that both deficiencies can be overcome by moving to a biologically more realistic encoding of each salient random variable through the stochastic firing activity of an ensemble of neurons. The resulting model demonstrates that networks of spiking neurons with noise can easily track and carry out basic computational operations on rapidly varying probability distributions, such as the odds of getting rewarded for a specific behavior. We demonstrate the viability of this new approach towards neural coding and computation, which makes use of the inherent parallelism of generic neural circuits, by showing that this model can explain experimentally observed firing activity of cortical neurons for a variety of tasks that require rapid temporal integration of sensory information.

## Author Summary

The Markov Chain Monte Carlo (MCMC) approach to probabilistic inference for a distribution  is to draw a sequence of samples from  and to carry out computational operations via simple online computations on such a sequence. But such a sequential computational process takes time, and therefore this simple version of the MCMC approach runs into problems when one needs to carry out probabilistic inference for rapidly varying distributions. This difficulty also affects all currently existing models for emulating MCMC sampling by networks of stochastically firing neurons. We show here that by moving to a space-rate approach where salient probabilities are encoded through the spiking activity of ensembles of neurons, rather than by single neurons, this problem can be solved. In this way even theoretically optimal models for dealing with time varying distributions through sequential Monte Carlo sampling, so called particle filters, can be emulated by networks of spiking neurons. Each spike of a neuron in an ensemble represents in this approach a “particle” (or vote) for a particular value of a time-varying random variable. In other words, neural circuits can speed up computations based on Monte Carlo sampling through their inherent parallelism.

## Introduction

**Figure 1:**
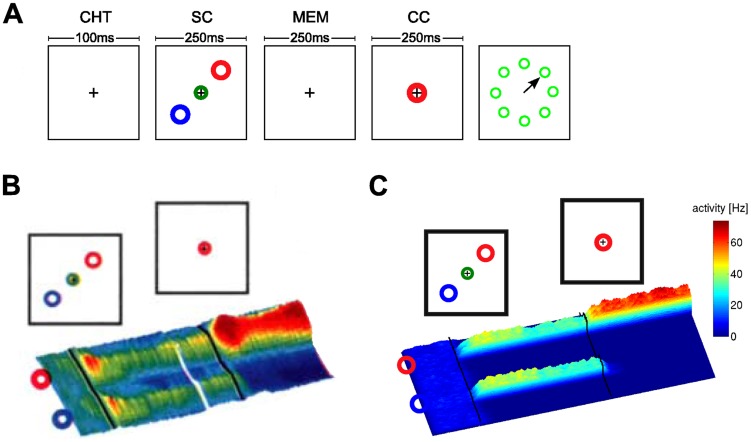
Representation of a belief in dorsal premotor cortex (PMd) in the ambiguous target task. **A)** Task structure. After an initial fixation (center-hold time; CHT), the spatial cue (SC) is shown in the form of two color markers at one of eight possible locations and displaced from each other by 180 degrees. They mark two potentially rewarded movement directions. After a memory epoch (MEM), the color cue (CC) is shown at the fixation cross. The rewarded movement direction is defined by the direction of matching color in the color cue (time periods of simulation indicated). **B)** Firing activity of neurons in dorsal premotor cortex during the task. Before the spatial cue is shown, neurons are diffusely active. As the spatial cue is shown, neurons with preferred directions consistent with the spatial cue increase the firing rate and others are silenced. This circuit behavior is retained during the memory epoch. As the color cue is presented, neurons with consistent preferred directions increase their firing rates. **C)** Simulation result for a circuit that performs evidence integration in ENS coding (activity smoothed; horizontal axis: time). Neurons are ordered by their preferred direction. Panel B modified with permission from [52].

Humans and animals are confronted with various situations where the state of some behaviorally relevant time-varying random variable 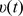 is only accessible through noisy observations 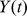. It is then essential to estimate the current value of that random variable 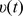, and to update this belief on the basis of further evidence. Over the last three decades we have learned from various experiments that monkeys are able to perform such operations. In the classical random-dot motion task, monkeys are confronted with dots on a screen moving in random directions, where a random subset of dots moves coherently. Monkeys are able to determine the direction of coherent motion even for low coherency levels [1]. A more recent study has shown that the firing rate of neurons in parietal cortex are proportional to the momentary log-likelihood ratio of a rewarded action for the given sensory evidence [2], suggesting that cortical circuits perform some form of probabilistic inference to determine the value of the hidden variable that represents rewarded actions. In yet another experiment, Cisek and Kalaska [3] studied macaque monkeys in an ambiguous target task. A visual spatial cue and a color cue, which were separated by a memory epoch, determined the rewarded direction of an arm movement, see Figure 1A. Ambiguity about the rewarded action after the first cue was reflected in the firing activity of dorsal premotor cortex (PMd) neurons, see Figure 1B. When the second cue determined the single rewarded action, only neurons tuned to the rewarded movement direction remained active. This finding suggests that estimates for the value of a salient time-varying random variable 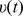 (getting rewarded for carrying out a specific action) are represented and updated through the current firing activity of different ensembles 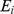 of neurons, one for each possible value 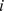 of the random variable 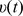.

We show that despite of their diversity, all these tasks can be viewed as probabilistic inference tasks, where some internal belief about the current value 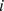 of a hidden random variable 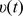 (e.g., which action is most likely to be rewarded at the end of a trial) needs to be updated based on often ambiguous sensory evidence 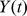 (moving dots, visual cues, etc.). We will distinguish 5 different classes of such tasks (labeled A - E in Results) that differ for example with regard to the time scale on which the hidden variable 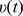 changes, or prior knowledge about the expected change of 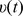. These tasks can not be solved adequately through a Hidden Markov Model (HMM). The reason is that a HMM generates at each moment in time just a single guess for the current value of an unknown variable. It is therefore not able to work with more complex temporary guesses, say that an unknown variable has probably value 1 or 2, but definitely not value 3. Obviously such advanced representations are necessary in order to make decisions that depend on the integration of numerous temporally dispersed cues.

For all these classes of computational tasks there exist theoretically optimal solutions that can be derived within a probabilistic inference framework. If one assumes that the hidden random variable 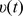 is static (i.e., 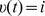 for some 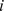 and all times 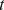), evidence provided by the temporal stream of observations has to be integrated in order to infer the internal belief about the value of the random variable 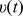. In such *evidence integration*, an initial prior belief formalized as a probability distribution 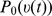 is updated over time in order to infer the time-varying posterior distribution 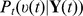, where 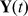 denotes all evidence up to time 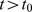. For example, an observation at time 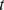 that is likely for 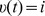 will increase the probability of state 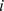 at time 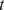, while the probability of values under which the observation is unlikely will be decreased. *Bayesian filtering* generalizes evidence integration to time-varying random variables. It is often assumed that the dynamics of the random variable is time-independent. Bayesian filtering then infers the posterior 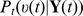 by taking the assumed dynamics of the time-varying random variable 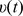 into account. For example, if value 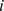 is currently likely, and state 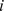 is likely to transition to state 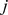, then the probability for state 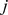 will gradually increase over time. For many important tasks, the dynamics of the random variable 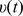 is not identical at all times but rather depends on context. For example, the change of body position in space (formalized as a hidden random variable 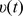) depends on motor actions. We refer to Bayesian filtering with context dependent dynamics as *context-dependent Bayesian filtering*. It infers 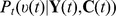, where 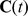 denotes all context information received up to time 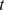.

In previous work, it was shown that networks of spiking neurons can embody a probability distribution through their stochastic spiking activity. This enables a neural system to carry out probabilistic inference through sampling (e.g., estimate of a marginal probability by observing the firing rate of a corresponding neuron) [4]. This model for probabilistic inference in networks of spiking neurons was termed neural sampling. However, neural sampling does not provide a suitable model for the representation and updating of quickly-varying distributions as it is needed for the tasks discussed above, since a good estimate of the current value of 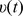 can only be read out after several samples have been observed. Another deficit of the aforementioned simple form of the neural sampling model is, that each salient random variable is represented through the firing activity of just a single neuron. This is unsatisfactory because it does not provide a network computation that is robust against the failures of single neurons. In fact, the representation of random variables through single neurons is not consistent with experimental data, see Figure 1B. In addition it requires unbiologically strong synaptic connections in order to ensure that the random variable that is represented by such single neuron has an impact on other random variables, or on downstream readouts. Furthermore, downstream readout neurons are required to integrate (count) spikes of such neuron over intervals of several hundred ms or larger, in order to get a reasonable estimate of the probability that is represented through the firing rate of the neuron (i.e., in order to estimate a posterior marginal, which is an important form of probabilistic inference).

We examine in this article therefore an extension of the neural sampling model, where random variables (e.g. internal beliefs) are represented through a space-rate code of neuronal ensembles. In other words, we are making stronger use of the inherent parallelism of neural systems. In this *ensemble based neural sampling (ENS) code*, the percentage of neurons in an ensemble 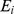 that fire within some short (e.g. 20 ms) time interval encodes the internal belief (or estimated probability) that a random variable currently has a specific value 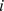.

This variation of the neural sampling model is nontrivial, since one tends to lose the link to the theory of sampling/probabilistic inference if one simply replaces a single neuron by an ensemble of neurons. We show however that ensemble based neural sampling is nevertheless possible, and is supported by a rigorous theory. In this new framework downstream neurons can read out current internal estimates in the ENS code just through their standard integration of postsynaptic potentials. We prove rigorously that this generates unbiased estimates, and we also show on what parameters the variance of this estimate depends. Furthermore we explore first steps of a theory of neural computation with the ENS code. We show that nonlinear computation steps that are needed for optimal integration of time-varying evidence can be carried out within this spike-based setting through disinhibition of neurons. Hence networks of spiking neurons with noise are in principle able to approximate theoretically optimal filtering operations – such as evidence integration and context-dependent Bayesian filtering – for updating internal estimates for possible causes of external stimuli. We show in particular, that networks of spiking neurons with noise are able to emulate state-of-the-art probabilistic methods that enable robots to estimate their current position on the basis of multiple ambiguous sensory cues and path integration. This provides a first paradigm for the organization of brain computations that are able to solve generic self-localization tasks. The resulting model is especially suited as “computational engine” for an intention-based neural coding framework, as proposed in [5]. Intention-based neural coding is commonly observed in lateral intraparietal cortex (area LIP) of monkeys, where neurons encode a preference for a particular target of a saccade within the visual field [1].

The remainder of this paper is structured as follows. First we introduce ENS coding. We then discuss basic properties of the ENS code. In *Computational operations through ensemble-based neural sampling*, we show how basic computations on time-varying internal beliefs can be realized by neural circuits in ENS coding. This section is structured along 5 classes of computational tasks of increasing complexity (*Task class A* to *Task class E*). Within these task classes, we present computer simulations where the characteristics of these neural circuits are analyzed and compared to experimental results. A discussion of the main findings of this paper and related work can be found in *Discussion*. Detailed derivations and descriptions of computer simulations are provided in *Methods*.

## Results

### Ensemble-based neural sampling

In neural sampling [4], each neuron in a network represents a binary random variable. Spike generation is stochastic, with a probability that depends on the current membrane potential. At each time 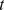, the activity of each neuron is mapped to a sample for the value of the corresponding variable by setting the value to 1 if the neuron has spiked in 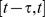 for some small 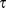 (e.g. 20 ms). It was shown that under certain conditions on the membrane potentials of the neurons, the network converges to a stationary distribution that corresponds to the posterior distribution of the represented random variables for the given evidence. Evidence is provided to the circuit by clamping the activities of a subset of neurons during inference. The marginal distribution for a given variable can be read out by observing the firing activity of the corresponding neuron in the stationary distribution.

Neural sampling is an implementation of the Markov Chain Monte Carlo (MCMC) sampling approach (see e.g. [6]) in networks of spiking neurons. By definition, it does not provide a suitable model for the representation of time-varying distributions, since samples are generated in a sequential manner. Convergence to the stationary distribution in MCMC sampling can take substantial time, the readout of marginal distributions demands spike counts of neurons over extended periods of time, and MCMC sampling is only defined for the fictional case of stationary external inputs. But also time-varying distributions can theoretically be handled through Monte Carlo sampling if one has a sufficiently parallelized stochastic system that can generate at each time point 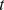 simultaneously several samples 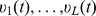 from the time varying distribution 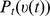, and by carrying out simple computational operations on this batch of samples in parallel. The resulting computational model is usually referred to as *particle filter*, a special case of sequential Monte Carlo sampling [6], [7]. Here each sample 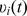 from a batch 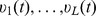 that is generated at time 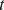 is referred to as a *particle*.

**Figure 2:**
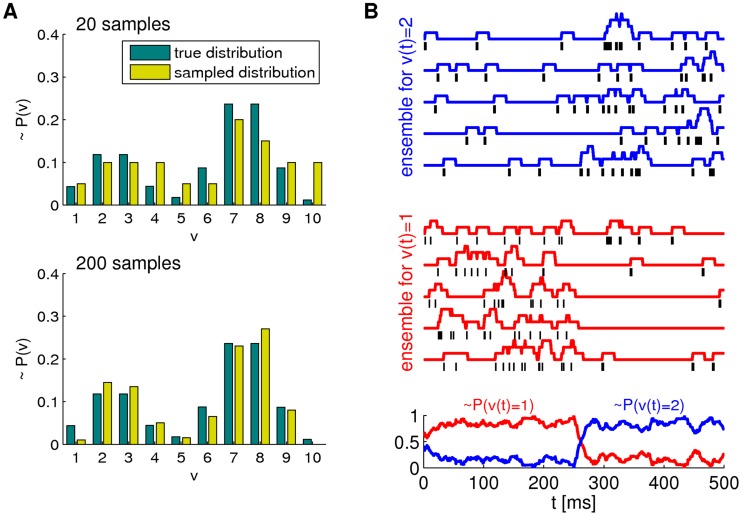
Spikes as samples from probability distributions. **A**) Sample-based representations of probability distributions. True distribution of a random variable 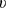 (green) and approximated distribution (yellow) based on 20 samples (top) and 200 samples (bottom) **B**) Interpretation of the spiking activity of two neuronal ensembles as samples from a probability distribution over a temporally changing random variable 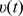. Shown is an example for a random variable 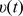 with two possible states. Black lines in the top traces indicate action potentials in two ensembles (5 neurons per state shown). Traces above spikes show EPSP-filtered versions of these spikes (red: state 1; blue: state 2). Bottom plot: Estimated probabilities for state 1 (red) and state 2 (blue) according to eq. (1) based on the spiking activity of 10 neurons per state.

To port the idea of neural sampling to the representation of time-varying distributions through neuronal ensembles, we therefore consider 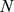 ensembles 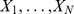 that collectively encode the belief about the value of a random variable 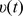 with range 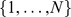 in terms of a probability distribution 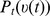. We refer to the value of a random variable also as the hidden state, or simply the state of the variable. We will in the following omit the subscript 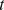 in 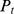 for notational convenience (formally, we consider a family of variables, indexed by 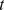, that defines a random process, see [8]; 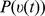 is then the distribution over the member 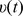 of this family). Each ensemble 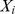 consists of 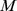 neurons 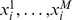, where we refer to 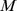 as the *ensemble size*. We interpret a spike in the circuit as one sample from 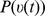, i.e., one concrete value for the represented variable drawn according to the distribution [4], [9]. In particular, if a spike is elicited by some neuron in ensemble 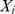, then this value is 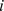. See Figure 2A for an illustration of sample-based representations.

A downstream neuron can evaluate how many spikes it received from each ensemble within its membrane time constant 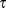 through summation of excitatory postsynaptic potentials (EPSPs) caused by spikes from ensemble neurons. We denote the EPSP-filtered spike train of neuron 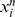 by 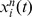 (see *Methods* for a precise definition) and adopt rectangular EPSP shapes of length 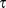 in the following, similar to those recorded at the soma of pyramidal neurons for dendrite-targeting synaptic inputs (see Figure 1 in [10]). The sum of all EPSPs from an ensemble 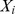, denoted by 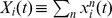, is then the number of samples for hidden state 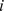 in the time window from 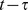 to 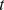. The number of samples 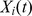 is thus directly accessible to downstream neurons. We refer to 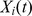 as the (non-normalized) probability mass for hidden state 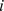. The use of plateau-like EPSP shapes is motivated from the need to count spikes in some predefined time interval. An alternative motivation that is based on the idea that the EPSP weights a spike by the probability that it belongs to the most recent samples is given in *Text S1*.

The represented distribution can be estimated by the relative portion of spikes from the ensembles
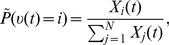
(1)where we assume that at least one sample is available. See Figure 2B for an intuitive illustration of ENS coding. In this representation, probabilities are temporally filtered by the EPSPs. Hence, the ability of the code to capture fast dynamics of distributions depends on the length of EPSPs, where shorter time constants give rise to faster tracking. Due to the stochasticity of the sampling process, 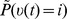 is a random variable that assumes different values each time the distribution is represented. We demand in ENS coding that the expected value of 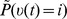 is equal to the temporally filtered represented probability 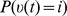 for all states 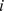, see *Methods* for details.

We will see in the construction of computational operations in ENS coding that downstream neurons do not have to carry out the division of eq. (1). Instead, for these operations, they can compute with the non-normalized probability masses 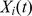 that they obtain through summation of EPSPs from ensemble neurons. The reason is that normalization is not necessary in the representation of a distribution in ENS coding. It is rather the relative portion of spikes for each value of the random variable that defines the represented distribution. Of course, activity needs to be kept in some reasonable range, but this can be done in a rather relaxed manner.

### Basic properties of the ENS code

Assume that samples (spikes) for state 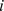 are produced by a Poisson process with rate proportional to the represented probability, i.e., the rates 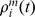 of neurons in ensemble 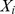 are given by 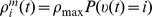, where 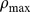 is a constant that defines the maximal instantaneous rate of each neuron. 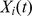 is then a random variable distributed according to a Poisson distribution with intensity 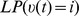, where the *estimation sample size* 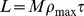 is the average number of spikes produced by all ensembles within a time span of 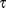.

We show in *Methods* that in this case, 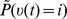 is an unbiased estimator of the probability of state 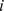 at time 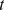 filtered by the EPSPs, hence, an ENS code is established. An important question is how parameters of a circuit influence the fidelity of the encoding. To answer this question, we investigated the variance of the estimator. It is inversely proportional to the ensemble size 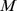, the maximal firing rate 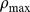, and the membrane time constant 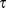 if the number of samples within 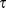 is large, see *Methods*. Hence, the accuracy can be increased by increasing the ensemble size, the firing rate of neurons, and the time constant of neuronal integration. Note however that an increase of the latter will lead to more temporal filtering of the distribution.

We close this discussion by considering the relation between the instantaneous firing rates of ensemble neurons and the mean represented probability mass 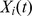. The probability mass is not an instantaneous function of the ensemble firing rate, since at time 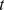 there are still past samples that influence 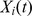 through their EPSPs. A past sample becomes invalid after time 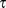, when the associated EPSP vanishes. The instantaneous firing rates *change* the mass through the production of novel samples. Consider given continuous firing rates 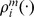. Under mild assumptions on the firing rates of ensemble neurons (see *Methods*), the change of the expected probability mass 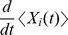 is then given by
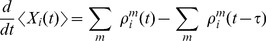
(2)where the expectation is taken over realizations of spike trains for the given instantaneous rates. Here, the first term is due to the production of novel samples and the second term due to old samples that become invalid. In summary, the membrane potentials of the neurons determine – through the firing rate – the rate of change of the represented probability mass in ENS-coding.

### Computational operations through ensemble-based neural sampling

We address now the question how basic computations on time-varying internal beliefs can be realized by neural circuits in ENS coding. Spiking activity of excitatory neurons is modeled according to the stochastic Spike Response model [11], [12]. In this model, each neuron 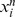 emits a Poisson spike train with instantaneous firing rate
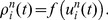
(3)


Here, 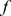 denotes a link function that links the somatic membrane potential 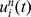 to the instantaneous firing rate. Typically, the link function is either an exponential function or a non-negative linear function. We consider in this article a non-negative linear link function 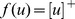 [12], where 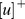 is 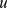 for non-negative 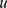 and 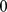 otherwise.

We discuss five classes of computational tasks.

#### Tasks class A

In these tasks, the state of a random variable 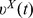 has to be inferred in ENS coding given the belief about a variable 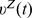 in ENS coding, where the distribution over 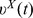 depends solely on the current state of 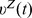. The computational operation needed to solve such problems is simpler than the other ones considered here in the sense that the distribution over 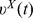 can be directly inferred from recent samples for 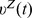. We will use this operation several times to read out the belief about a rewarded motor action (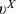) from the internal belief about some random variable 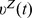.

#### Tasks class B

This class consists of tasks that can be solved through evidence integration. In other words, the state of a random variable 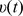 has to be estimated based on evidence 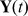, where 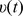 is assumed to be static during each trial. Examples for such tasks are the ambiguous target task, the random-dot motion task, and the probabilistic inference task from [2]. We will exhibit a spiking neural network architecture that approximates optimal solutions for these tasks in ENS coding and compare its behavior to experimental results.

#### Tasks class C

Also in these tasks, evidence 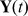 has to be integrated to estimate the state of a random variable 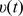. However, the state of 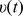 may change over time according to known time-independent stochastic dynamics. Bayesian filtering provides an optimal solution for such tasks. We will extend the circuit architecture from task class B to approximate Bayesian filtering in ENS coding and test its performance in a generic task setup.

#### Tasks class D

In this class of tasks, the dynamics of the random variable 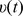 may change during the task, and changes are indicated by some context-variable 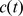. Note that task classes B and C are special cases of this task class. We refer to the optimal solution as context-dependent Bayesian filtering. An approximation based on sequential Monte Carlo sampling is particle filtering. We will extend our circuit architecture to perform full particle filtering in ENS coding. We will show how the important problem of self-localization can be solved by this architecture.

#### Tasks class E

Finally, we will discuss tasks where the context variable 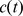 is not explicitly given but has to be estimated from noisy evidence. Hence, 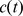 – which determines the dynamics of 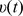 – has also to be considered a random variable. We will treat such tasks by combining two particle filters. One particle filter estimates 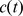 and provides context for another particle filter that estimates 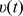. As an example, we will reconsider the ambiguous target task. We show that a belief about the current stage within a series of trials can be generated in order to decide whether evidence should be further integrated for the belief about the rewarded action or whether a new trial has started and the belief should be reset to some prior distribution.

### Task class A: Simple probabilistic dependencies

We first discuss how the belief for a random variable 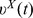 can be inferred in ENS coding given the belief over a variable 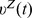. Consider a random variable 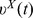 for which the distribution depends solely on the current state of a random variable 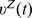. The task is to infer the distribution over 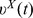 for the current distribution over 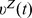. This operation is needed for example in typical decision making tasks where inference about a rewarded action has to be performed according to the belief about some hidden variable that is based on sensory information. For example, the rewarded movement direction has to be guessed, based on the belief about the perceived cue combination in the ambiguous target task.

Assume that 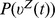 is constant and represented by neurons 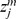 through ENS coding with estimation sample size 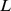. We are looking for a neural circuit that represents the posterior belief
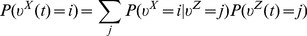
(4)in ENS coding. Here, 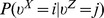 are the known conditional probabilities that determine the dependencies between 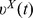 and 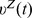.

**Figure 3:**
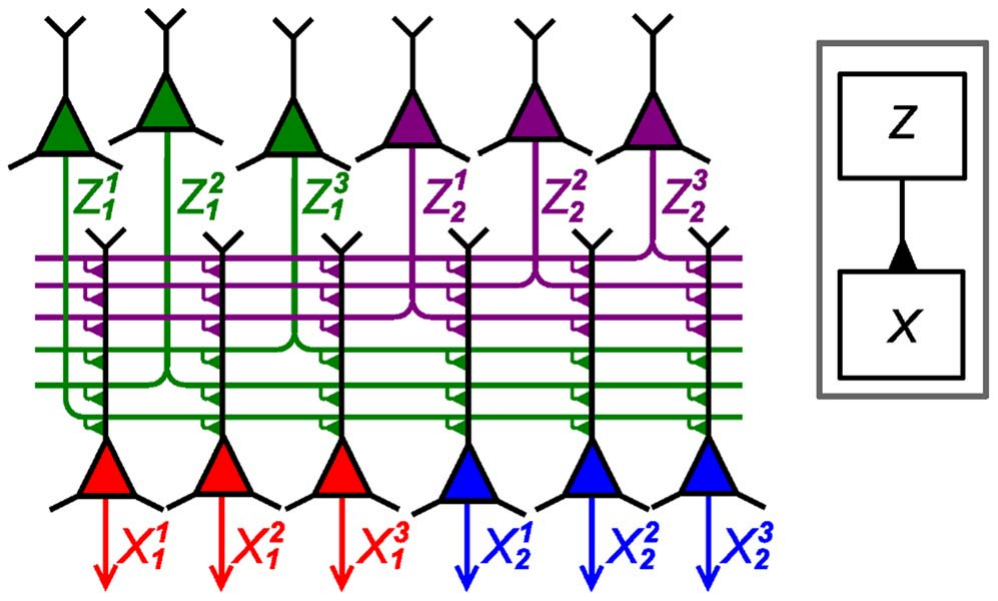
Computations in ENS coding in a feed forward circuit architecture. A binary random variable 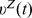 is represented in ENS coding through neurons 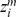. The posterior 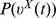 for a binary variable 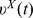 is represented by neurons 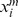. Each variable is represented by 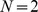 ensembles, one for each possible state (indicated by neuron color), and 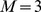 neurons per ensemble. The two layers are connected in an all-to-all manner. Arrows indicate efferent connections (i.e., outputs in ENS coding). The architecture is summarized in the inset.

We consider a layer of ensembles 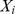 that receive feed-forward synaptic input from ensembles 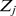 representing 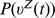, see Figure 3. The membrane potentials of neurons 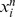 are given by
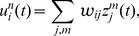
(5)where 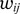 denotes the efficacy of the synapse connecting presynaptic neuron 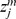 to postsynaptic neuron 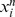 (we assume for simplicity of notation that all weights between two ensembles are identical). Consider the estimator 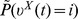 for synaptic efficacies
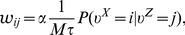
(6)where 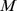 is the ensemble size of the ensembles 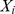, and 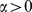 is some constant. Due to the stochastic nature of neurons, this estimator is a random variable. Its expected value (with respect to realizations of spikes trains in all ensembles) is equal to the posterior probability and the estimation sample size is 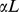 (see *Methods*). Thus, the layer represents the posterior distribution in ENS coding. The estimate at some specific time 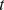 is however variable due to variability in spike counts of both the representation of 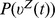 and the representation of 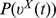. An analysis of the variance of the posterior representation is given in *Methods*.

We will use such a layer with feed-forward input several times in our simulations to infer a belief about rewarded actions for a given belief about the state of a random variable and thus refer to it as an *action readout layer*.

We will also need a special case of this operation where the distribution 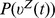 is simply copied, i.e., the state of the random variable 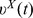 is assumed to be identical to the state of 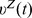. In other words, 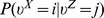 is 1 for 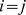 and 0 for 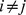. The copy operation is thus performed for weights 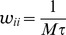 and 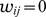 for 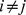 (where we used 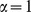).

### Task class B: Evidence integration

In pure evidence integration, the value of the random variable 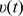 is assumed to be constant and only indirectly observable via stochastic point-event observations. Point-event observations are assumed to arise according to Poisson processes with instantaneous rates that depend on the current hidden state. In the context of neuronal circuits, observations are reported through spikes of afferent neurons 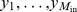. In particular, afferent neuron 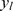 is assumed to spike in a Poissonian manner with rate 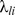 if the hidden variable assumes state 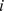. For a prior distribution over states 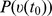, the task is to infer the posterior 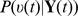, that is, the distribution over states at time 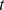, given the spike trains of all afferent neurons up to time 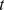, denoted here by 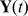. Many laboratory tasks can be formalized as evidence integration task, including the random-dot motion task, the probabilistic inference task considered in [2] and the ambiguous target task discussed in the introduction. We construct in the following a circuit of spiking neurons that approximates optimal evidence integration by performing particle filtering in ENS coding under the assumption that 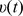 is constant. The will then evaluate its behavior against experimental data in computer simulations. This circuit will be extended in the subsequent sections to perform particle filtering for tasks classes C and D.

It is well-known that the evidence integration problem can be solved efficiently through a set of coupled differential equations
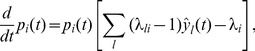
(7)where 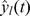 is the spike train of afferent neuron 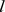 formalized as a sum of Dirac delta pulses at spike times (see *Methods*), and 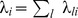 [13], [14]. The inferred probabilities can be obtained by normalization 
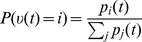
 (for 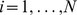). 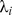 induces a constant decrease of 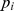 such that hidden states that give rise to many observations are punished if no observations are encountered. Is the dynamics (7) compatible with ENS coding, assuming that 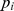 is estimated from the spiking activity of an ensemble? Four potential difficulties arise. First, the afferent neurons impact eq. (7) via point events and not via EPSPs (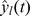 instead of 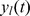). Second, the deterministic dynamics (7) need to be implemented via particles in the ENS code. Third, the summed evidence needs to be multiplied with the current value of 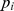. And finally, to avoid an exponential blow-up of the 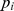
's, their values need to be normalized. We discuss these four issues in the following.

#### Evidence can be provided through EPSPs

It turns out that the first difficulty can be resolved in a convenient manner. The set of differential equations (7) can be transformed to
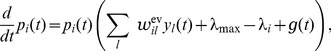
(8)with weights 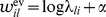 for an arbitrary constant 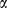, 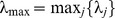, and an arbitrary function 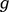. Integration of EPSPs weighted by 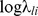 leads to exactly the same result as integration of eq. (7) after the EPSPs have fully been integrated, even if they are temporally overlapping, see *Methods*. The constant 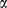 can be used to shift 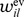 to positive values. This constant, 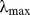, and the function 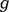 are integrated by all 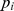
's giving rise to a scaling that cancels in the normalization.

#### Particle-based implementation of the filtering equations

**Figure 4:**
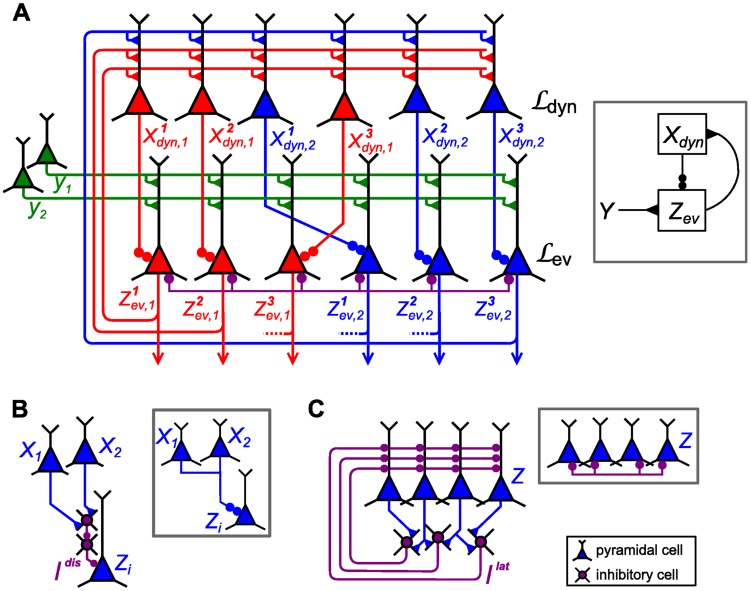
Particle filter circuit architecture for task classes B and C. **A**) Circuit with 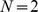 ensembles (indicated by red and blue neurons respectively) and 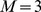 neurons per ensemble. Neurons in layer 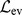 receive synaptic connections from neurons in layer 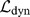 and update the represented distribution according to evidence input from afferent neurons (green). Lateral inhibition (magenta; see panel C) stabilizes activity in this layer. Neurons project back to layer 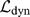. For task class B (evidence integration; static random variable 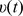), only connections between neurons that code for the same hidden state are necessary and layer 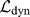 simply copies the distribution represented by layer 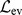, see *Task class A* and Figure 3 (in contrast to Figure 3, the copying ensembles 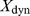 are plotted above ensembles 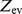 in order to avoid a cluttered diagram). For task class C (Bayesian filtering; random variable 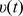 with time-independent dynamics), 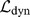 implements changes of the represented distribution due to the dynamics of the random variable and 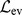 is potentially fully connected to 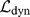. Neurons in layer 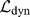 disinhibit neurons in layer 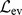 (double-dot connections; see panel B). Disinhbition and lateral inhibition is indicated by shortcuts as defined in B, C. Arrows indicate efferent connections. A schematic overview of the circuit is shown in the inset. **B**) Disinhibition 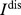: neurons 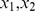 excite an interneuron (purple) which inhibits the inhibitory drive to some neuron 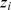. As a graphical shortcut, we draw such disinhibitory influence as a connection with two circles (inset) **C**) Lateral inhibition: Pyramidal cells (blue) excite a pool of inhibitory neurons (magenta) which feed back common inhibition 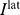. The graphical shortcut for lateral inhibition is shown in the inset.

We now discuss how eq. (8) is approximated in ENS coding. While eq. (8) is a deterministic differential equation, the dynamics of the circuit is stochastic due to sampling noise. We construct a circuit that approximates the desired changes in its expected probability masses. The validity of this approach will later be ascertained through various computer simulations. The belief about the hidden state of the random variable 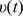 is in general shaped by two components. First, the assumed internal dynamics of the random variable, and second by novel evidence about the state. Consider a circuit that consists of two layers 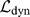 (the dynamics layer) and 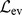 (the evidence layer) with neural ensembles 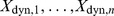 and 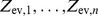 respectively, see Figure 4A. The dynamics layer 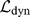 implements changes of the represented distribution due to the internal dynamics of the random variable. The evidence layer 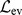 implements changes due to incoming evidence. Since in task class B, the random variable is assumed to be static, no temporal changes of the random variable are expected, and hence the excitatory weights 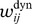 from 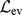 to 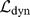 are set such that 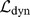 copies the distribution represented by 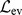, as discussed in *Task class A*. For task classes C and D, different weights will be used such that layer 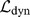 predicts dynamics changes of the hidden variable.

The evidence layer 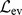 receives input from 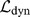 and evidence from afferent neurons 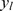. Our goal is that its probability masses integrate evidence in the representation of 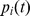 in 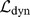, such that
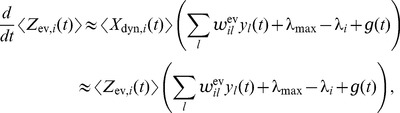
(9)where the latter approximation applies due to the copy operation of layer 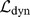. This equation resembles eq. (8) where the 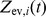
's represent the 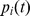
's. We show in *Methods* that such changes are obtained if the membrane potentials of the neurons in 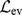 are set to
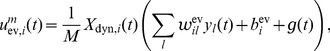
(10)where 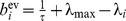 are positive biases.

#### Multiplication through gating of activity

We see that neurons need to compute a multiplication between the current probability mass for state 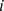 and the summed evidence. In order to implement similar equations in a neuronal-like manner, logarithmic dendritic nonlinearities or multiplicative synaptic interactions have been postulated in a number of studies, see e.g. [14], [15]. In ENS coding however, the population response in the evidence layer is the sum of the responses of individual neurons. This linearity allows us to base the membrane potential of an individual neuron 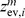 on a small number of particles rather than on the whole set of particles summarized in 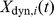, as long as each particle is used exactly once in the computation. Hence, the same behavior is obtained on the population level if instead of membrane potentials (10), membrane potentials are given by
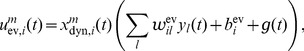
(11)see *Methods* for a detailed derivation. If spiking of individual neurons 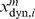 is sparse, i.e., if the ensemble size is large compared to the estimation sample size, 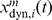 nearly always takes on the values 0 or 1. In this case, it suffices that the activity of neuron 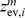 is *gated* by neuron 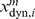, i.e., the neuron 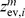 is able to produce spikes only if 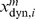 was recently active. In summary, under the assumption of sparse activity (which can always be accomplished by a suitable choice of parameters), one can replace the multiplication of two analog variables by gating of activity in ENS coding. This multiplication strategy generalizes the one proposed in the context of stochastic computation to ensemble representations [16], [17].

Such gating could be accomplished in cortical networks in various ways. One possibility is synaptic gating [18], [19] where inputs can be gated by either suppression or facilitation of specific synaptic activity. Another possibility is disinhibition. Disinhibitory circuits provide pyramidal cells with the ability to release other neurons from strong inhibitory currents [20]. We choose in this article disinhibition as the gating mechanism, although no specific mechanism can be favored on the basis of the experimental literature. A small circuit with disinhibition 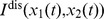 is shown in Figure 4B. Here, two pyramidal cells excite an interneuron which inhibits the inhibitory drive to some neuron 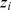. Functionally, 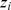 is released from strong baseline inhibition if one of the neurons 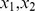 was recently active (see *Methods* for a formal definition). Using disinhibition, the membrane potential of the neuron can be written as
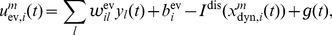
(12)where 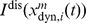 ensures that the firing rate 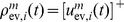 is nonzero only if neuron 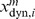 did spike within the last 20 ms.

#### Stabilization of firing rates through lateral inhibition

Lateral inhibition is generally assumed to stabilize the activity of excitatory populations [21]–[23]. A group of pyramidal cells inhibit each other laterally by projecting to a group of inhibitory neurons which in turn inhibit that ensemble, see Figure 4C. The key observation that enables us to use lateral inhibition to stabilize circuit activity is that one has freedom to choose 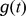 in eq. (12) as long as it is identical in all ensembles. We model lateral inhibition 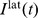 that depends on the recent firing activity in 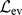 such that inhibitory activity increases if the estimation sample size is above the desired value 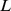 and choose 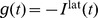, see *Methods* for details and a brief discussion. This concludes the construction of a particle filtering circuit in ENS coding for task class B. The circuit architecture is depicted in Figure 4A. A summary of circuit equations can be found in Table 1.

**Table 1:** Particle filter circuit equations for task classes B and C.

Layer	Ensembles	Neurons	Membrane voltage and parameters
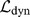	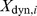	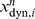	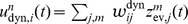
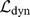	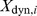	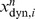	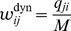 for 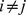 , 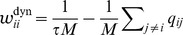 for all *i*.
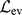	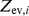	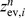	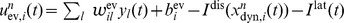
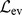	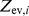	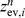	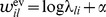 , 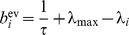

Here we have defined 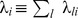 and 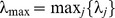. 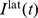 denotes lateral inhibition and 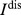 disinhibition. 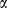 is an arbitrary constant. In task class B (evidence integration), 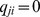 for 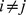, leading to 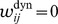 for 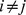 and 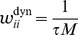.

**Figure 5:**
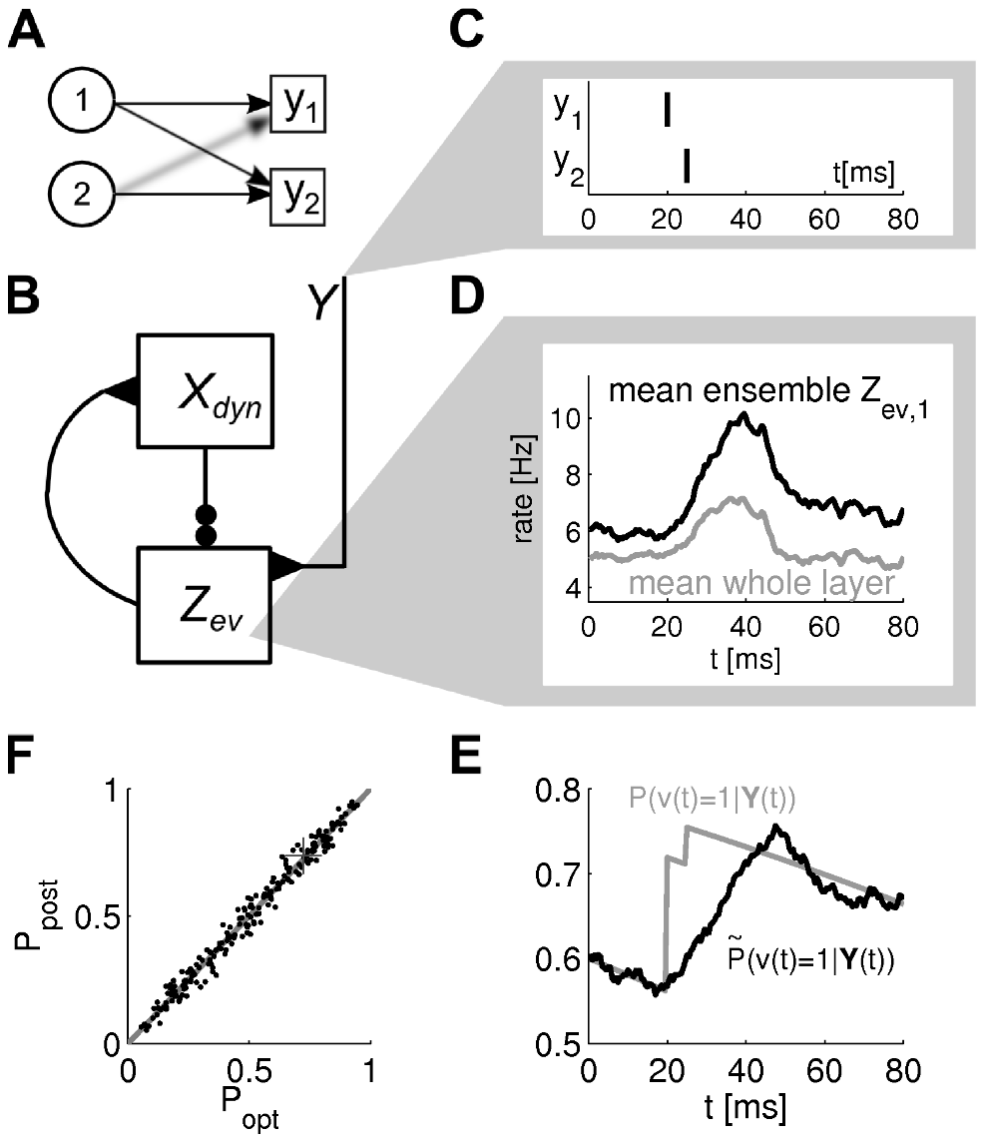
Evidence integration through particle filtering in ENS coding. **A**) The state of a binary random variable 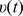 that gives rise to two possible observations 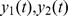 is estimated. Both observations occur more frequently in state 1 (indicated by sharpness of arrows). **B**) Estimation is performed by a particle filtering circuit with evidence input 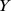 (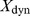: dynamics layer ensembles; 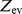: evidence layer ensembles). **C**) An evidence spike is observed at times 20 ms and 25 ms in evidence neuron 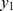 and 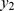 respectively. **D**) Example for the rate dynamics in layer 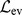. Ensemble rate for ensemble 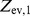 (black) and whole layer 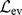 (gray). The input leads to a transient increase in the ensemble rate. Inhibition recovers baseline activity. The ensemble rate for state 1 undergoes a transient and a sustained activity increase. **E**) Temporal evolution of estimated posterior probability 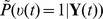 for state 1 (black) in comparison to true posterior 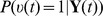 (gray) for this example run. **F**) Posterior probability at *t* = 45 ms (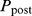) for state 1 of the circuit in comparison to true posterior at this time (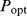). Each dot represents one out of 100 runs with prior probabilities and observation likelihoods drawn independently in each run (see *Methods*). The results of the example run from panels A–E is indicated by a cross.

We tested how well a circuit consisting of 2000 neurons per state and an estimation sample size of 400 can approximate the true posterior in a simple evidence integration setup. The task was to compute the posterior distribution for a random variable 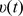 with two hidden states and two observable variables 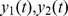, see Figure 5A. The schematic circuit diagram is shown in panel B. The two evidence neurons 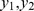 spiked at times 20 ms and 25 ms respectively, see panel C. Figure 5D depicts the rate dynamics in layer 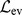 for one example trial. Novel evidence transiently increases the firing rate in the layer, which is in turn restored by lateral inhibition. The response of ensemble 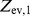 that represents state 1 undergoes a transient increase that is counteracted by inhibition until it stabilizes at an enhanced sustained level. This behavior is reminiscent of the typical response of cortical pyramidal cells to sensory input. Figure 5E shows the temporal evolution of the encoded posterior probability 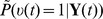 in comparison to the true posterior 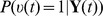. The true posterior is approximated very well after a delay of about 20 ms, which is the time needed to integrate the EPSPs from evidence neurons. We simulated 100 trials where in each trial, prior probabilities for the states and observation likelihoods were drawn randomly such that the posterior 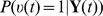 at time *t* = 45 ms assumed values between 0 and 1 (see *Methods*). The estimate of the circuit at the end of the second EPSP (i.e., at time *t* = 45 ms) is shown in comparison to the true posterior in Figure 5F.

Gating of activity for multiplication can be used for many types of multiplicative operations on probability distributions. In *Text S2*, we discuss its application to cue combination, an operation that has been considered for example in [24].

### Comparison to experimental results

We performed computer simulations in order to compare the behavior of the model to various experimental studies on tasks that are examples for task class B.

#### The ambiguous target task

**Figure 6:**
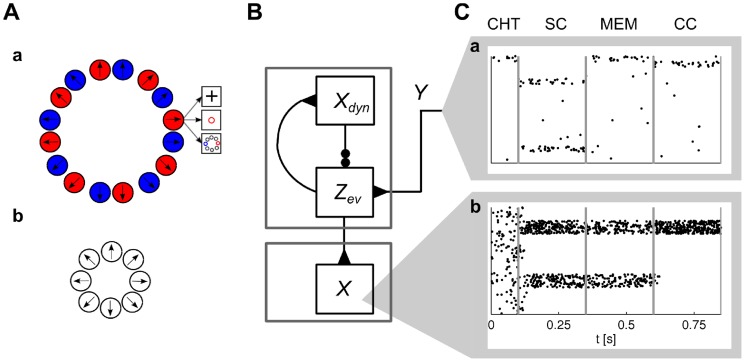
Particle filtering in ENS coding for the ambiguous target task. **A**) Represented random variables. **Aa**) Evidence integration is performed for a random variable with 16 hidden states corresponding to direction-color pairs. Values of the random variable are depicted as circles. Observations accessible to the monkey in one example state are shown as boxes. **Ab**) The action readout layer infers a color-independent random variable by marginalization over color in each direction. **B**) Circuit structure. The circuit on the top approximates evidence integration through particle filtering (top gray box; 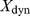: dynamics layer ensembles; 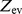: evidence layer ensembles)) on the random variable indicated in panel (Aa). An action readout layer (bottom gray box; ensembles *X*) receives feed-forward projections from the particle filter circuit. **C**) Spike rasters from simulations for afferent neurons (Ca) and neurons in the action readout layer (Cb). Each line corresponds to the output of one neuron. Afferent neurons are ordered by feature selectivity (e.g., top neurons code the presence of the fixation cross). Action readout neurons are ordered by preferred movement direction. See also Figure 1.

The ambiguous target task studied in [3] was already discussed above, see also Figure 1A. In our model of the decision making process, the hidden state of a random variable 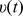 was estimated through evidence integration. Each of the 16 hidden states corresponded to a tuple 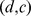, where 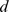 denotes one of eight possible directions of movement, and 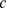 denotes the color of the color cue. In other words, such a state represented the color of the color cue and the movement direction that leads to reward, see Figure 6Aa. Possible observations were the fixation cross, the spatial cues at 8 positions in two colors, and the two color cues. Each of the 19 possible stimuli was coded by 20 afferent neuron that fired at a baseline rate of 0.1 Hz. When a stimulus was present, the corresponding neurons spiked in a Poissonian manner with a rate of 5 Hz.

We simulated a particle filter circuit to compute the belief about the state of the random variable with 1000 neurons per hidden state and an estimation sample size of 400. An action readout layer as described in *Task class A* was added that received connections from 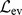 in a feedforward manner, see Figure 6B. This layer computed the current belief over rewarded actions independently from the color of the color cue (i.e., it marginalized over color), see *Methods* for details.

The spiking activity of afferent neurons that provided evidence for one example simulation run is shown in Figure 6Ca. Simulated neural activities from the readout layer are shown in Figure 6Cb, see also Figure 1C. After the spatial cue was presented, the two consistent ensembles increased their activity. Due to competition between these ensembles, neurons fired at a medium rate. After the color cue was shown, only the ensemble consistent with both the spatial and the color cue remained active. These neurons increased their firing rate since the competing action became improbable and the winning ensemble was uncompeted. This behavior has been observed in PMd [3], see Figure 1B. The action readout layer is not needed to reproduce this behavior, since neurons in the particle filter circuit exhibit similar behavior. However, it was reported that most neurons in PMd were not color selective [3]. In our model, neurons of the particle filter circuit are color selective since states are defined according to direction-color pairs. It is clear that color-related information has to be integrated with movement-related information and memorized during the memory epoch in order to solve the task. The experimental results suggest that this integration is not implemented in PMd but rather in upstream circuits. PMd could then act as a motor readout.

Applications of the model to various other experimental tasks can be found in supporting texts. Action-predictive activity in macaque motor cortex is also modulated by the expected value of the action. This was demonstrated for example in [25]. An application of our model to this scenario is described in *Text S3*. Furthermore, we show in *Text S4* that the model is consistent with features of neuronal activity during random-dot motion tasks [1], [26], [27]. In *Text S5* it is shown that the model can also explain neuronal activitiy in area LIP during a probabilistic reasoning task [2].

### Task class C: Bayesian filtering

Evidence integration cannot take temporal changes of the hidden variable into account. Knowledge about temporal changes can be exploited by Bayesian filtering, which is an extension of evidence integration. Here, we assume that the dynamics of 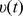 are constant during the filtering process. The more general case when the dynamics may change is discussed below in *Task class D*. Formally, the Bayesian filtering problem considered here is to estimate the posterior distribution 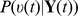 over the states of a random variable 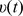 that represents the hidden state of a random process which is only indirectly observable via stochastic point-event observations 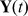. In particular, state changes are assumed to be Markovian with *transition rates* 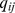 for each pair of distinct states 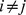. Transition rate 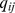 defines the rate of transition from state 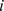 to state 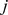, i.e., the probability that a transition occurs to state 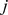 in some small time interval if the current state is 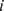, thus defining a continuous time Markov chain, see *Methods* for a more formal description.

Note that evidence integration is a special case of Bayesian filtering with the assumption of no state transitions, i.e., 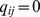 for all 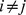. In the following, we show that the particle filtering circuit for task class B constructed above can easily be extended to this generalization. The Bayesian filtering problem can be solved efficiently through a set of coupled differential equations
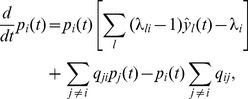
(13)where again the inferred probabilities are obtained by normalization [13], [14]. Note that the first term in eq. (13) is identical to eq. (7), since the optimal solution for evidence integration, eq. (7), is the special case of eq. (13) for vanishing transition rates. This term is taken care of in layer 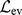 of the particle filtering circuit for task class B. In this circuit, 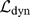 simply copies the distribution given by 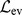. We modify the connections from 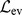 to 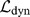 such that 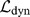 instead provides the changes in probability masses needed for the second and third term, i.e., it predicts changes based on the assumed dynamics of the random variable. 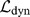 approximates the desired changes of probability masses if the membrane potentials are given by
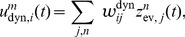
(14)with synaptic efficacies 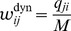 for 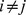 and 
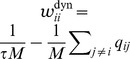
 for all 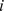, see *Methods*. An overview of the circuit equations and parameters is given in Table 1, see also Figure 4A.

**Figure 7:**
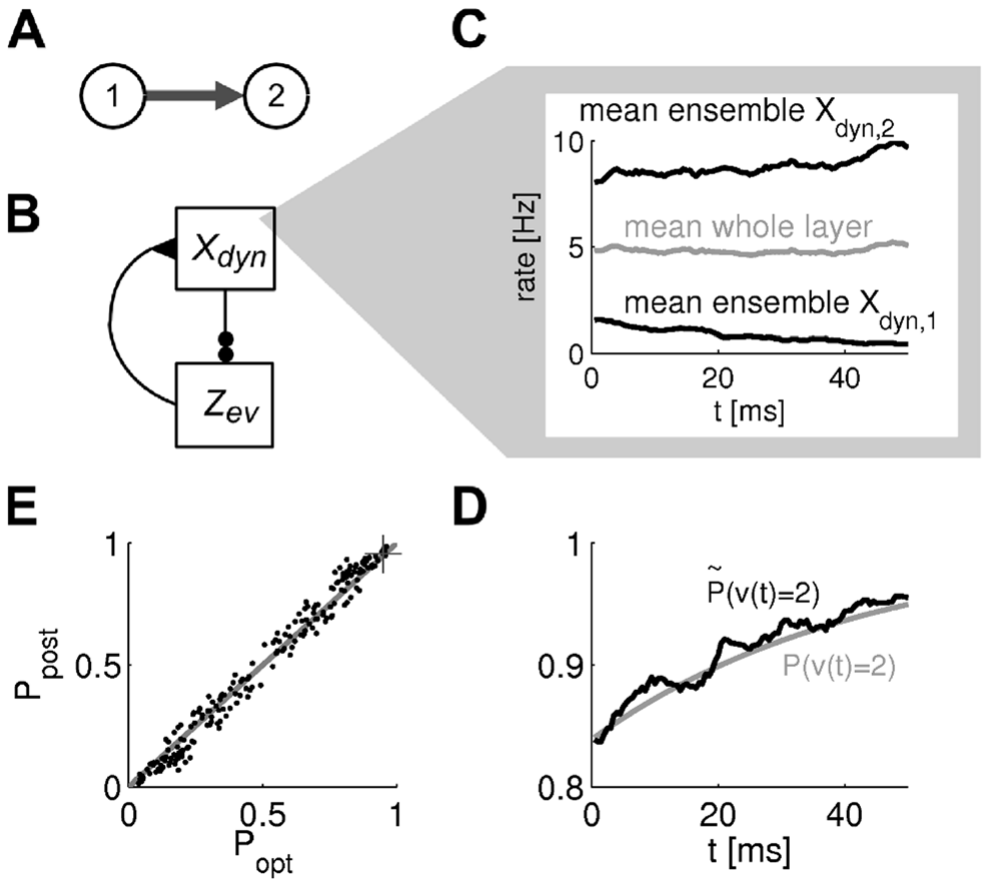
Tracking of dynamics in ENS coding. **A**) The state of a binary random variable 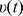 is estimated where state 1 transitions to state 2 with some transition rate 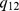. **B**) Estimation is performed by a particle filter circuit without evidence input (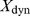: dynamics layer ensembles; 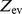: evidence layer ensembles). **C**) Example for the rate dynamics in layer 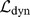. Ensemble rate for ensembles 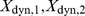 (black) and whole layer 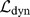 (gray). While rates in ensembles change due to the prediction of a transition, inhibition keeps the overall firing rate in the layer approximately constant. **D**) Temporal evolution of estimated posterior probability 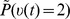 for state 2 (black) and true posterior 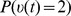 (gray) for this example run. **E**) Circuit estimates of posterior probabilities at time *t* = 50 ms (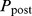) in comparison to true posteriors at this time (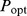). Shown are 100 runs (dots) with prior probability for state 1 and transition rate drawn from uniform distributions in [0.1, 0.9] and [0, 30]Hz respectively in each run. The result of the example run from panels C-D is indicated by a cross.

We tested the ability of a particle filter circuit consisting of 2000 neurons per state and an estimation sample size of 400 to track the temporal evolution of a binary random variable where state 1 transitions to state 2 with some transition rate 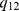, see Figure 7A. The dynamics of the circuit and the estimated probability for an example simulation run are shown in panels C,D. We simulated 100 trials, where in each trial the transition rate 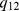 was drawn uniformly in [0, 30]Hz and initial probabilities were drawn uniformly in 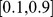. The estimate of the posterior at time *t* = 50 ms is shown in comparison to the true posterior in Figure 7E. See *Methods* for details on this simulation.

#### Particle filtering in a generic setup for task class C

**Figure 8:**
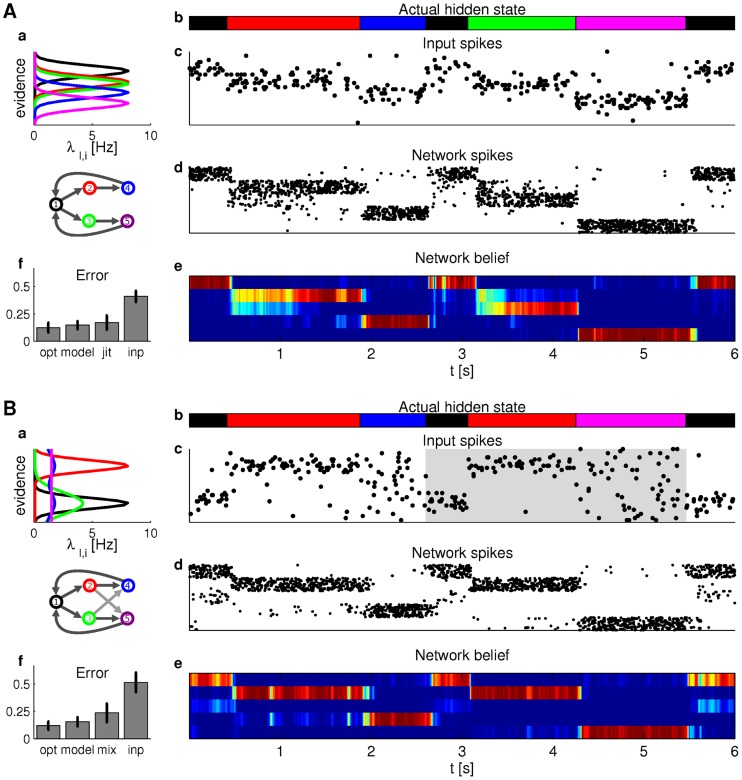
Particle filtering for task class C (A) and task class D (B) in the ENS code. **Aa**) State diagram of the Markov chain for the dynamics of the hidden random variable (bottom) and state-dependent firing rates of afferent neurons (top). Colors indicate the value of the hidden state. **Ab**) Actual hidden state over time is indicated by color in correspondence with colors in panel Aa. **Ac**) Spike trains of afferent neurons. Each line corresponds to the output of one afferent neuron ordered according to panel Aa. **Ad**) Network response to the input in panel Ac. Neurons are ordered according to their preferred state from state 1 (top neurons) to 5 (bottom neurons). **Ae**) Network belief (estimated posterior state probability) derived from network activity. Rows ordered by state as neurons in panel Ad. Hot color indicates high probability of the state. Note the uncertainty when state 2 or 3 is entered. **Af**) Summary of network performance (“model”; fraction of incorrect state estimates) in comparison with the optimal Bayesian filter (“opt”), a network with jittered synaptic efficacies (“jit”), and the optimal decision based on the most recent observation only (“inp”). Bars are means and errorbars STDs over 20 state and observation sequences (12 seconds each). **B**) Particle filtering for task class D. **Ba**) As panel Aa but with context. Dark gray arrows in the state diagram indicate transitions in context A. In context B, the transitions from states 2 to 4 and 3 to 5 are interchanged (light gray arrows). **Bb**) As panel Ab. Background shading indicates context (context A: white; context B: gray). **Bc–Be**) Actual hidden state, input spikes, networks spikes, and network belief; see panels Ac–Ae. **Bf**) Summary of network performance. “opt” shows performance of the optimal context-dependent Bayesian filter and “mix” a Bayesian filter where the transition rates are the mean rates over contexts A and B. The spiking network performs significantly better than the mixed Bayesian filter (paired t-test, *p*<0.001).

We performed further computer simulations in order to test the performance of the model in a generic setup. In this setup, a random variable 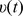 evolved according to a continuous time Markov chain with five states. In this Markov chain, state 1 transitions to states 2 or 3 which themselves transition to states 4 and 5 respectively. From states 4 and 5, a transition to state 1 is possible, see Figure 8Aa (bottom). Transition rates for all possible transitions were set to 1Hz. Information about the actual state was conveyed by 35 afferent neurons with state-dependent rates defined by Gaussians as shown in Figure 8Aa (top). Note that states 2 and 3 gave rise to very similar observations. Thus, many observations have to be integrated before these states can be distinguished. This makes inference over the current state hard if the full distribution over state probabilities is not communicated over time.

Results from simulations of a particle filter circuit with 2000 neurons per state and an estimation sample size of 400 are shown in Figure 8Ac–Af, see *Methods* for details. Figure 8Af shows a comparison of the model performance (“model”) with the optimal Bayesian filtering (“opt”) and the optimal model that does not take temporal information into account (the Bayes estimate based on the latest observation; “inp”). The performance of the model was very close to optimal and much better than the non-temporal Bayes estimate. We furthermore tested the robustness of the network to variations of synaptic efficacies 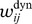 that determine the assumed transition rates of the random variable. In a control experiment, each individual synaptic weight from ensemble 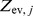 to ensemble 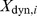 was drawn from a log-normal distribution with mean 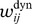 and standard deviation 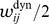. The resulting weights assumed values that were up to 10 times larger than the mean. Network performance with jittered efficacies was indistinguishable from the performance of the homogeneous network (Figure 8Af; “jit”). This robustness stems from two features of ENS-coding. First, as network belief is represented by ensemble rates, it is invariant to firing rate variations of individual neurons as long as the ensemble rate is preserved. Second, network computations are generally based on averages over ensemble activities, see eq. (14). Therefore variations in synaptic efficacies do not influence the result as long as the mean ensemble-to-ensemble weights are preserved. Note that this even holds for nonlinear link-functions, eq. (3).

### Task class D: Context-dependent Bayesian filtering

In many important situations, the dynamics of a random variable changes in different contexts. Context cannot simply be formulated as a type of observation since observations just influence the probabilities of states for the given transition rates and not the dynamics themselves. Consider for example the estimation of the current body position in space. Here, an action such as forward movement can be considered as context since it increases the transition rates from any position to positions ahead. Thus, it changes the transition rates and not just the probability of a particular position. For 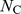 possible contexts, consider a function 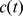 with range 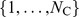 that indicates the context at time 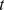. We define a context-dependent Markov chain as a Markov chain with state transition rates 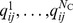 for each pair of distinct states 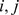. At each time 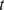, the chain evolves according to 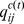. Context-dependent Bayesian filtering determines the distribution over the current state for the given observations and context. Note that Bayesian filtering used in task class C is a special case with just a single context. Also note that we use the term *context* here with the specific meaning of additional information about the dynamics of the random variable.

**Figure 9:**
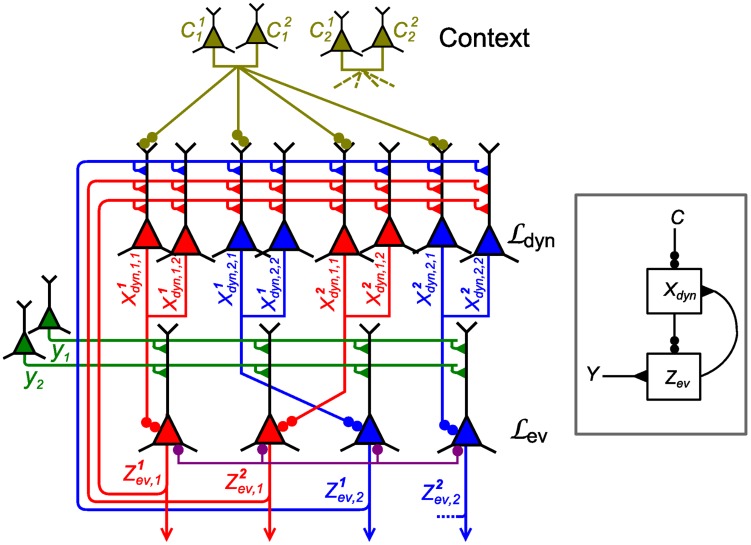
Particle filter circuit architecture for task class D. Extended circuit with 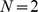 ensembles (indicated by red and blue neurons respectively) and 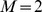 neurons per ensemble and two possible contexts. Ensembles in layer 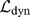 are duplicated for each context. These neurons receive context information via disinhibition from context neurons (yellow; only connections from context 1 shown for clarity). Disinhbition and lateral inhibition indicated by shortcuts as defined in Figure 4B, C. Arrows indicate efferent connections. A schematic overview of the circuit is shown in the inset.

We encode the current context by ensembles 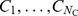 that provide contextual feedback to the circuit. Each context ensemble 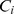 consists of 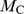 context neurons 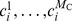. At time 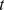, only the context ensemble 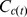 is active. The circuit architecture for particle filtering (Figure 4A) is extended as shown in Figure 9 to perform particle filtering for a hidden random variables 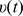 with context-dependent dynamics. Layer 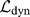 consists of 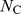 representations of the random variable, one for each context. Hence, for each context 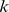 there are 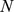 ensembles 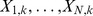 in this layer. In addition to excitatory synaptic connections originating from 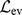, these neurons are disinhibited by context neurons from the corresponding context ensembles. The disinhibitory effect of context is consistent with experimental data about neocortical circuits. There, context information from other cortical areas is believed to be provided through feedback connections. Those connections have abundant terminals in neocortical layer 1 where they recruit disinhibitory circuits [28], [29]. Formally, the membrane potential of 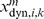, the *m*-th neuron in ensemble 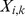, is given by
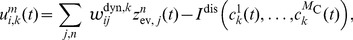
(15)where 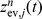 denotes the EPSP-filtered spike train of a neuron in ensemble 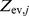 of 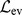 and 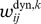 denotes the efficacy of the connecting synapse. These efficacies are set proportionally to the transition rates in the corresponding context, see Table 2. The membrane potential of a neuron in 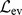 is similar to the non-contextual case with the difference that each neuron is disinhibited by neurons that code for the same state in various contexts
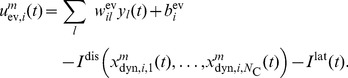
(16)


**Table 2:** Particle filter circuit equations for task class D.

Layer	Neurons	Membrane voltage and parameters
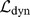	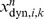	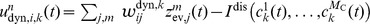
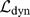	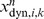	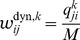 for 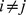 , 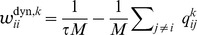 for all *i*.
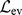	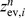	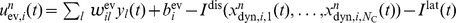
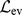	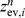	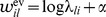 , 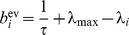

Here we have defined 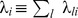 and 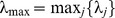. 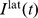 denotes lateral inhibition and 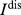 disinhibition. 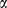 is an arbitrary constant.

Due to disinhibition of layer 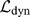 by context ensembles, only those ensembles in the layer which correspond to the current context are active. The active neurons in this layer disinhibit neurons in 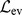 in the same way as in the non-contextual case. Thus, in each individual context, a subcircuit is recruited that consists of the whole layer 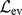 and the ensembles for the current context in layer 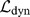. Since the weights 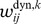 from 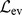 to ensembles for context 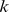 in 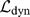 implement the dynamics of the random variable in that context, the circuit approximates the correct transition dynamics of the random variable in each context. The resulting membrane potential equations are summarized together with parameters in Table 2.

#### Particle filtering in a generic setup for task class D

In order to test the ability of the model to perform context-dependent Bayesian filtering, we considered a random variable with context-dependent dynamics. Two possible contexts A and B were indicated by 20 context neurons. The underlying Markov chain with context-dependent transition rates is shown in Figure 8Ba (bottom). The dynamics in context A was equivalent to the one considered in the generic test for task class C (dark gray arrows). However in context B, state 2 exclusively transitioned to state 5 and state 3 exclusively to 4 (light gray arrows). Additionally, we considered a more complex observation model in this example (panel Ba, top). State-dependent firing rates were either Gaussians with varying variances, bimodal, or uniform. Note that states 4 and 5 gave rise to quite similar observations. Hence, it is hard to distinguish these states without context. Panels Bc-Be show that the model makes good use of this context information (context indicated by gray shading in panel Bc). At time *t* = 2 s, there was a transition from state 2 to state 4 in context A, and at time *t* = 4.25 s, there was a transition from state 2 to state 5 in context B. In both cases, the network estimate followed immediately since the expected transitions were modulated by context information. We also tested the performance of an optimal context-dependent Bayesian filter and a Bayesian filter without context information. This filter was based on a dynamics model with mean transition rates over both contexts, which resulted in suboptimal performance, see Figure 8Bf. Implementation details are given in *Methods*.

#### Particle filtering in ENS coding for self-localization

Estimation of the body position in space (self-localization) is an essential ingredient of autonomous agents. In robotics, particle filtering is one of the most successful techniques for self localization [6]. Here, we demonstrate that particle filtering in ENS coding can be used for self-localization in environments with ambiguous evidence.

**Figure 10:**
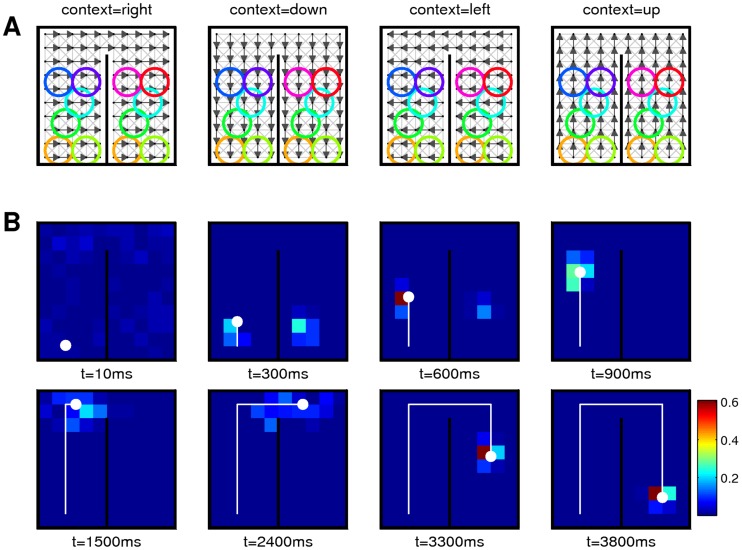
Self-localization through particle filtering in ENS coding. **A**) Two-chamber maze (black lines) and transitions between states in various contexts. States are arranged on a 10×10 grid in the maze (crossing points of gray lines). Light gray lines indicate bidirectional state transitions with low transition rates (0.1 Hz). Dark gray arrows indicate transitions with high transition rates (3.5 Hz). Context is defined by movement direction (right, down, left, up). Colored circles indicate sensory evidence. Each color stands for one afferent neuron with a Gaussian spatial receptive field. The circle indicates the STD of the Gaussian. Note that the southern chambers give rise to identical observations. Observations are truncated at the height of the opening between the chambers such that no observations are experienced in the most northern parts. **B**) Network estimate of posterior probability (see color bar on the right for color code) for one trajectory through the maze (white trace; dot denotes current position) at different times. Spatial layout as in A. Various phases of the trajectory are shown: Uninformative prior knowledge (*t* = 10 ms), ambiguous estimates (*t* = 300 ms); disambiguation (*t* = 600, 900 ms); states without evidence (*t* = 1500, 2400 ms); unambiguous state estimation based on ambiguous evidence (*t* = 3300, 3800 ms).

Every state of the considered hidden random variable corresponded to some position in the environment and transitions were possible between spatially adjacent states. The movement of the agent provided context for the particle filter such that movement in a particular direction enhanced transitions that point to that direction (Figure 10A). Sensory cues provided partial information about the current position. We simulated a two-chamber maze with a small opening that connects these chambers. The southern parts of the chambers gave rise to exactly the same observations, making it impossible to distinguish them without prior information (colored circles in Figure 10A). Observations in more northern parts were different and in the very north, no observations were experienced (corresponding for example to a dark corridor). Figure 10B shows the estimate of the network for a single trajectory. The model was started with a uniform prior distribution over all positions. In the southern terrain, the left and the right chamber cannot be distinguished, and accordingly, ensembles in both areas were active, indicating possible positions (*t* = 300 ms). In the more northern parts, observations disambiguate the current position, and activity in the right chamber ensembles ceased (*t* = 600, 900 ms). As the upper northern region was reached, no more evidence was provided from the environment (*t* = 1500, 2400 ms). Still, the network predicted movement to the right correctly thus utilizing movement information provided via context ensembles. As the right chamber was entered, the posterior was sharpened due to unambiguous sensory input. It remained single peaked even in southern parts of the chamber although these terrains produced ambiguous sensory input (*t* = 3300, 3800 ms).

### Task class E: Internal beliefs as context

In many tasks, the context variable 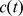 is not explicitly available to the animal but rather has to be estimated from noisy evidence 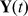 as well. For example, in many sequential tasks the current stage within the task can provide valuable context information which can be used to time actions or to decide when beliefs should be reset. In the computational framework considered here, this can be achieved by treating the context variable 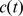 as a random variable that is estimated by a particle filter circuit from the given evidence 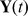. Estimated context is then utilized for the estimation of a random variable 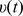 in a particle filter circuit with context.

In the tasks considered in task class D, the context was unambiguously given and all neurons of exactly one context ensemble had a firing rate larger than zero. This is not the case in task class E, since here context neurons encode a belief about the current context. The context-dependent particle filter should therefore deal with context in a graded manner. This is accomplished by a simple modification of how neurons in layer 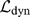 integrate context, see *Methods*. In the modified circuit, effective transition rates 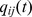 of the context-dependent filtering circuit are given as a linear mixture of the context-dependent transition rates 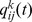, where each contributes approximately proportionally to the current belief 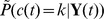 in this context
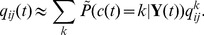
(17)


To demonstrate the viability of this approach, we reconsidered the ambiguous target task.

#### The ambiguous target task revisited

**Figure 11:**
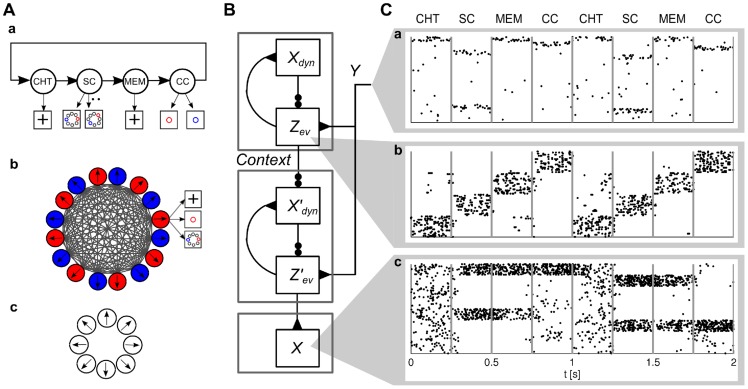
Context-dependent Bayesian filtering in two successive trials of the ambiguous target task. **A**) Represented random variables. **Aa**) Dynamics of a random variable that codes the current phase in a trial of the ambiguous target task (CHT: fixation; SC: spatial cue; MEM: memory cue; CC: color cue). Possible observations in each phase are indicated in boxes. **Ab**) Context-dependent Bayesian filtering is performed for a random variable with 16 hidden states corresponding to direction-color pairs as in Figure 6Aa. Gray lines indicate context-dependent transitions. All-to-all transitions are possible in the fixation phase (CHT). There are no transitions in other phases of a trial. **Ac**) The action readout layer infers a color-independent random variable by marginalization over color in each direction. **B**) Circuit structure. The circuit on the top (ensembles 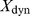 and 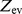) performs Bayesian filtering on the random variable 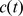 indicated in panel (Aa). It provides context for another particle filter circuit (middle gray box; ensembles 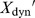 and 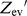) that generates a belief about the random variable 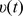 indicated in Ab. An action readout layer is added (bottom gray box; ensembles 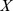). **C**) Spike rasters from a simulation of two successive trials for afferent neurons (Ca), neurons in the particle filter circuit for the phase in the trial (Cb), and neurons in the action readout layer (Cc). Neurons in Cb are coding for the current phase of the trial (ordered from bottom to top: CHT, SC, MEM, and CC). Neuron ordering in Ca and Cc as in Figure 6.

The ambiguous target task has a clear sequential structure (initial fixation, spatial cue, memory epoch, color cue), see Figure 1A. The corresponding hidden variable *c*(*t*) – for which the current value is given by the momentary stage of the experimental trial – is shown in Figure 11Aa.

The modeled task is similar to the ambiguous target task considered above with the difference that several trials of the task are performed sequentially, i.e., a sequence of epochs (fixation, spatial cue, memory, color cue) is directly followed by the fixation epoch, indicating the start of a new trial, again followed by the spatial cue and so on. The difficulty of this task is that in the fixation epoch, the internal belief about the random variable 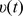 that encodes the current color-direction pair is highly biased by the last trial. This bias is problematic since an optimal prior would assign equal probability to all color-direction pairs. In other words, during the fixation, 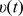 should be reset. A reset can be accomplished by assuming that during fixation the dynamics of 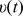 are such that 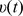 can quickly change its state from any state to any other state (i.e., transition rates are high between all pairs of states, see Figure 11Ab). In other epochs however, the assumption is that the value of the random variable is fixed and does not change (transition rates are zero between all pairs of distinct states). We arrive at a context-dependent Bayesian filtering problem where the dynamics of 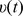 depend on the current estimate of 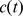, the epoch in the trial.

We modeled the context-dependent reset of the internal belief by extending the circuit for the ambiguous target task in the following manner. The internal belief about the random variable 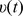 that encodes the current color-direction pair (Figure 11Ab) is generated by a particle filter circuit. Context is provided to this circuit by an estimate of 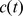, the current epoch in the trial (Figure 11B). This estimate is performed by a particle filter circuit that receives the same sensory evidence but no context (task class C). In the context of the fixation-epoch, 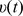 is believed to change its state rapidly (all-to-all transitions in the dynamics of the random variable, gray connections in Figure 11Ab). In the context of other epochs, the random variable is believed to be constant (no transitions between states, i.e., pure evidence integration). Finally, an action readout layer derives the belief over the rewarded action. In Figure 11C, a simulation for two successive trials of the ambiguous target task is shown. The epoch 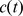 is correctly inferred by the particle filter circuit that provides the epoch-context (Figure 11Cb). This context influences information processing in the subsequent circuit for 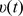 (Figure 11Cc). While information is retained during the memory epochs, the belief about the rewarded movement direction returns to an uninformative prior during the memory epoch at 1 s to 1.25 s. This example demonstrates that internally generated beliefs about random variables can act as valuable context in ENS coding.

## Discussion

It has recently been demonstrated that the dynamics of recurrent networks of spiking neurons can perform MCMC sampling on a distribution 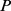 [4], [9]. The distribution can be approximately recovered by observing the evolution of the network state trajectory for some time. Such a temporal representation is less suitable for distributions that have to be updated rapidly since each estimate of 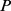 needs several hundreds of milliseconds. We have therefore proposed and analyzed in this article ENS coding, where ensembles of neurons code for each state of a random variable. In this coding scheme, adaptations of internal beliefs can be established on the time-scale of EPSPs, see Figures 5E and 8. Similarly, downstream readout neurons can rapidly estimate ENS coded probability values according to eq. (1) from neural ensembles, while such readout operation from neural sampling networks demands the integration of spikes over intervals of several hundred milliseconds. Another deficiency of the neural sampling approach is the need for unbiologically strong synaptic connections. This results from the principle that random variables are encoded by single neurons in neural sampling, which necessitates strong connections in order to ensure sufficient impact on postsynaptic targets. Unbiologically strong synaptic connections are not necessary in ENS coding, since targets can be activated in a cooperative manner by neuronal ensembles. This is for example apparent in the inverse scaling of weights with ensemble size in eq. (6).

We have shown that particle filtering [6], [7] can be performed by circuits of spiking neurons in ENS coding. Numerous engineering applications of particle filtering to tasks belonging to task class D exist. In such tasks, particles are evolved according to a dynamics-model that depends on context, such as the movement direction in a self-localization task. This particle filtering with context cannot be emulated by approximate Bayesian filtering as described in task class C. To the best of our knowledge, this article provides the first proof that this powerful operation is in principle accessible to spiking neural circuits. We have demonstrated in computer simulations that ENS coding enables neuronal circuits to perform these essential operations with high fidelity, thus making them suitable for higher-level decision-related processing. Lee and Mumford [30] proposed that particle filtering could be the basic computational operation in hierarchical cortical processing. We have demonstrated a first step in that direction in a spiking model with ENS coding by showing that the belief about a random variable can provide context information for the temporal processing related to other variables, see *The ambiguous target task revisited*.

### Model simplifications and possible extensions

For consistency with the neural sampling approach, we used in this article rectangular shaped EPSPs. To test how deviations from rectangular EPSPs effect circuit performance, we performed simulations where all EPSPs were modeled as exponentially decaying EPSPs with decay time constant 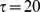
ms such that the integral over the EPSP is unchanged. The particle filter circuit was tested in the generic setup for task class C (see Figure 8A). Despite of its strong deviation from the rectangular shape, exponential EPSPs caused only a slight decrease of circuit performance (the percentage of incorrect state estimates was 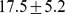
% as compared to 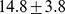
% for rectangular EPSPs).

The model also uses particular forms of instantaneous lateral inhibition and disinhibition. Although both forms of inhibition have been reported to play crucial roles in cortical information processing [20]–[23], [28], [29], the exact circuitry and function of those inhibitory circuits is still unknown. The lateral inhibition used in our circuit model is however consistent with a recent study which showed that inhibition has a broader spatial selectivity than excitation in visual cortex of awake mice [31]. Such broader selectivity is expected in our model since lateral inhibition is common to all ensembles for a random variable. Apart from that, the assumption that inhibition follows excitation instantaneously is clearly a model simplification. A recent experimental study [32] revealed that cortical inhibition lags excitation by about 3 ms in anesthetized rats (the lag is possibly smaller in awake animals, see [31]). In order to test whether the circuit is tolerant to delayed inhibition, we performed control simulations where in addition to the use of exponential EPSP shapes, both lateral inhibition and disinhibition was delayed by 3 ms in the generic setup for task class C (see Figure 8A). We found that delayed lateral inhibition can lead to activity peaks as excitation arising from incoming evidence cannot be compensated rapidly. This can lead to unstable circuit activity when lateral inhibition tries to compensate these peaks in the delayed negative feedback loop. Instabilities can be avoided by reduction of the inhibitory drive, i.e., by reducing the lateral inhibition scaling 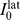 in eq. (33). Simulations showed that with reduced inhibition scaling, the network tolerates delayed inhibition with a slight decrease of performance (the percentage of incorrect state estimates was 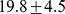
% as compared to 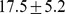
% without delay; 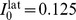).

In ENS coding, each neuron is tuned to one value of a random variable 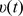. The encoded random variable may represent a specific feature relevant for some task. Many experiments show that such tunings exist in various cortical areas, such as the tuning of PMd neurons to potentially rewarded movement direction [3]. However, the random variable 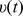 does not necessarily correspond to a single task-relevant feature. For example, the random variable encoded in the particle filter circuit for the ambiguous target task represents direction-color pairs (see Figure 6Aa). Therefore, neurons in this circuit are selective for both the spatial cue and the color cue. This mixed tuning helps to integrate the temporally separated cues. Mixed selectivity of neurons has been found in higher cortical areas such as prefrontal cortex, and its computational benefits have been highlighted in [33]. Hence, ENS coding is consistent with these findings. We note however that in the pure formulation of ENS coding, mixed selectivity to many task aspects is problematic since the number of ensembles necessary to encode all possible states over 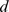 task dimensions grows exponentially with 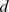. One possibility to overcome exponential growth for large 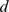 is to consider approximations schemes such as neglecting mixed configurations that are highly unlikely.

The important question how the parameters of the network could be attained by learning from experience is outside of the scope of this paper. However, some possible solutions to the learning problem can be sketched. For the particle filtering circuit, two classes of synaptic connections could be adapted through learning processes. First, synaptic efficacies 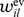 from evidence neurons to neurons in layer 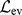. These efficacies encode the log-firing rates of evidence neurons for the given hidden state 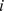. It has been shown that hidden-cause representations that require such synaptic efficacies can be learned in spiking neural networks with lateral inhibition through spike-timing-dependent (STDP)-like synaptic plasticity rules [34], [35]. Hence, it seems quite feasible that the efficacies 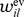 can be attained in an self-organized manner through STDP. The second type of connections, 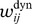 from 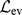 to 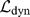 encode the dynamics of the random variable in terms of the rate of change 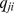 from state 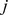 to state 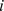. In other words, the synapse needs to track how often neuron 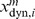 is active after neuron 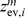 (the synaptic efficacies needed for task class A are of a similar nature). Again, this tracking can be done by a temporal Hebbian learning rule. In particular, it has recently been shown in [36] that such temporal relationships can be learned through STDP-like learning rules in networks of spiking neurons that implement hidden Markov models. A more sophisticated learning approach that requires additional circuitry was outlined in [37]. Of course, these results do not immediately generalize to the architecture proposed in this article, and further studies are needed to prove the viability of such a learning approach. Finally, we note that the feature of the particle filter circuit (and ENS coding in general) that individual synaptic efficacies do not need to be adjusted to exact values as long as the mean efficacy between ensembles is correct (see *Particle filtering in a generic setup for task class C*) may prove advantageous for learning processes.

### Experimentally testable predictions

We investigated the behavior of the model and compared it to experimental results. Our results so far indicate that ENS coding is consistent with a number of experimental studies. It is noteworthy that the lateral inhibition that is needed to stabilize network firing rates leads to the typical transient ensemble rate increases at stimulus-onset (see transient responses in Figure 5D and Figure 1C in *Text S5*).

While many laboratory tasks implement a variant of evidence integration (task class B), there is a lack of studies in the experimental literature for task classes D and E. We hypothesized in *Task class E: Internal beliefs as context* that the current values of important context variables are estimated in higher brain areas. Hence, one prediction of the model is that neural activities in such areas should not only be related to variables of primary interest, but also to context such as the current phase in a task with sequential structure, see Figure 11B,C. Evidence for such representations in monkey dorsolateral prefrontal cortex has been reported [38]. Our model also predicts that activity in neuronal ensembles that represent context should modulate the activity of decision-related ensembles in a manner that is fundamentally different from the impact of direct evidence. In particular, according to our circuit models in task classes D and E, context gates or modulates activity in such neurons. Task context has been shown to modulate neuronal activity in primate prefrontal cortex [39] as well as in various lower level visual areas including area MT [40], [41]. These findings are contrasted by studies that showed that task-context influenced noise-correlations but not firing rates in MT in a variant of the random-dot motion task [42]. The general term context of course subsumes many different types of contextual information in various quite different task settings, which may explain the discrepancies between the different studies. In this work, context is defined specifically as additional information about the dynamics of the random variable. Experimental setups where context information is indicative of the dynamics of task-relevant variables would help to elucidate how such information alters temporal processing in cortical circuits.

We proposed disinhibition as one possibility for context-dependent modulation, consistent with the experimental findings that disinhibitory circuits are recruited by feedback connections in neocortical layer 1 [28]. We note however that such effects could be implemented in cortical circuits by a number of mechanisms [18], [19].

We applied particle filtering – one of the most successful techniques for self localization in autonomous robots – in ENS coding to self-localization, a particularly important task for many animals. Whether self-localization in animals is solved in a similar manner is of course still unknown. There have however been studies which show that ambiguous sensory information can be resolved on the neuronal level in rodents [43], [44]. This indicates that the algorithm employed by the brain is in fact quite powerful. The particular implementation proposed here also implies that the spatial structure of the environment (i.e., possible transitions between locations in space) should be encoded in the synaptic weight matrix of particular neural circuits (edges and arrows in Figure 10A). Furthermore, our model predicts that information about motor events (movement) is treated by such circuits as context. Hence, these signals impact circuit activity quite differently from sensory evidence, see the discussion above. Evidence for nonlinear interaction of visual information and movement information in self-localization of mice has been reported recently [44].

### Related work

Probabilistic population codes (PPC; [24]) have been suggested as one hypothesis how probability distributions could be coded in the spiking activity of neurons. In the PPC concept, one assumes that each neuron is (at least implicitly) linked to a stimulus via a tuning function. A hypothetical decoder would then apply Bayes rule to decode the stimulus distribution, making use of the tuning functions of the neurons. In ENS coding considered here, the neural ensembles produce samples from a distribution and a hypothetical decoder would just count spikes. Accumulation of evidence in LIP in a random-dot motion task has been modeled in the PPC framework [45]. In general, information can be accumulated in PPC simply by adding up activity from afferent neurons, given that this activity follows Poisson-like statistics. The model however assumes that the hidden variable is static. In fact, tracking of dynamic variables, such as those considered in task classes C–E, is hard to implement in the PPC framework [46].

Several spiking neural network models for Bayesian filtering have been proposed in the literature with very similar basic ideas to solve the problem [15], [47], [48]. Denève [47] proposed a model where a single integrate-and-fire neuron estimates the hidden state of a binary random variable with temporal dynamics. The model can only deal with binary variables, whereas our proposed model is not restricted in this respect. A similar model was proposed in [48]. There the assumption was that the continuous-valued random variable evolves according to a drift-diffusion process. Our implementation of Bayesian filtering in ENS coding complements this work by considering discrete-valued random variables with the assumption that the dynamics can be approximated by a continuous-time Markov chain.

Rao [15] considered Bayesian filtering in discrete time through a network of spiking neurons. This model was however not based on a rigorous coding scheme with respect to information transfer through spikes. The instantaneous firing rate of individual neurons was regarded as the distribution-encoding quantity and it was implicitly assumed that spikes can communicate this quantity in sufficient quality. In the current article, we base representations of beliefs on the spiking activity in the first place and propose ENS coding as a solution where the fidelity of representation is provided through ensemble activity. Our analysis identified how network properties such as the ensemble size, the maximal firing rate, and the membrane time constant influence the quality of the representation. In any case, the noise introduced by stochastic spiking cannot be neglected in general. We have demonstrated in computer simulations that still, temporal information processing on demanding tasks is possible in ENS coding.

We have argued that particle filtering with context is an important operation that is needed for example for self-localization. The current work shows that this extension of Bayesian filtering, that has not been considered in previous models, can easily be implemented in the ENS code.

Several non-spiking models for Bayesian filtering have been proposed previously [14], [49], [50]. We have based our circuit model for task classes B and C on well-known filtering equations [13] that also provided the basis for the rate-based model considered in [14]. Several conclusions can be drawn when comparing the model based on ENS coding considered here with the non-spiking model from [14]. First, we confirmed through computer simulations that quite demanding information processing tasks are possible with spiking neurons using ENS coding, despite of substantial noise introduced by stochastic spiking. Second, ensemble coding is clearly necessary for the tasks considered in this article if neuronal responses are stochastic. We used on the order of 1000 neurons per ensemble in the simulations. This number was not optimized, but ensemble sizes below 100 are not sufficient, for example in the ambiguous target task (see *Dataset S1*). Third, besides the complications that stochastic spike codes introduce, we have shown that ENS coding also has some positive effects. We found that multiplicative operations can be replaced by gating of neuronal activity in ENS coding, for example through synaptic gating or through disinhibition. This property of ENS coding provides an attractive alternative to previously proposed solutions for the unavoidable demand of nonlinear processing in Bayesian filtering, such as multiplicative interaction of synaptic inputs [14], [50], or the use of precise dendritic nonlinearities [15]. Finally, the use of a spiking model enabled us to directly compare model characteristics to experimental data. We found that the model is consistent with quite diverse experimental results [1]–[3], [25].

### Conclusions

Sample-based representations of probability distributions provide an attractive framework for modeling probabilistic inference on static evidence in cortical networks. We have shown that ensemble-based neural sampling enables cortical networks to perform also powerful context-dependent temporal inference. Hence, our model provides a new and theoretically founded basis for understanding temporal probabilistic computations in various higher-level cortical areas.

## Methods

For easy reference, Table 3 summarizes the notational conventions used in this article.

**Table 3:** Notation.

Variable name	Description
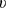	Random variable with range 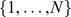 .
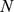	Number of states of 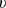 and number of ensembles that represent 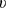 .
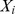	Ensemble *i* represents the belief that a random variable is in state *i*.
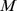	Number of neurons per ensemble.
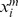	*m*-th neuron in ensemble 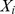 .
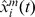	Spike train of neuron 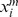 at time *t*.
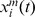	EPSP-filtered spike train of neuron 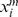 at time *t*.
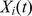	Summed activity (probability mass) for state *i* at time *t*: 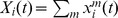 .
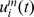	Membrane potential of neuron 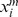 at time *t*.
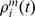	Instantaneous firing rate of neuron 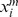 at time *t*.

Description of frequently used variables for easy reference. In general, capital letters refer to ensembles and lower case letters to neurons in these ensembles.

### Spike trains, EPSP shapes, and EPSP-filtered spike trains

Here, we define EPSP-filtered spike trains and the shape of EPSPs used throughout the article. We denote the spike-train 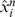 of a neuron 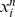 as the sum of Dirac delta functions
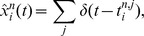
where 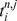 denotes the *j*-th spike-time of neuron 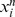. We define the EPSP-filtered spike train 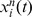 as



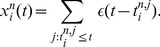
(18)Here 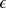 denotes the EPSP shape produced by a spike of a presynaptic neuron. We use in this article rectangular EPSP shapes of the form
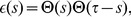
(19)where 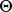 denotes the Heaviside step function and 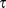 is the length of the EPSP. For some control simulations, we use exponentially decaying EPSP shapes


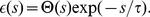
(20)

### ENS code and filtered probability distributions

Assuming rectangular EPSP shapes of length 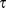, the filtered probability denoted as 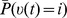 is the average probability for state 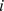 in time 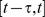, that is
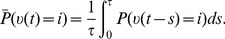
(21)


We denote the ensemble average of a random variable by 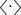. Consider a given fixed temporal evolution of the probability 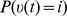. In ENS coding, we demand that neural activities are such that the mean of the estimator 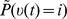 is equal to the temporally filtered probability of that state at time *t*, that is
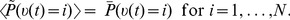
(22)


### Mean and variance of the estimator

Here we show that eq. (1) is an unbiased estimator of the probability of state 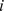 at time 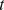 filtered by the EPSP, given that there is at least one spike in the integration window. Additionally we compute the variance of the estimator.

Consider a given fixed temporal evolution of the probability 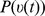. We first consider the mean of 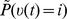 for a given number of spikes 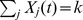, written as 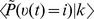:
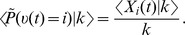



Given a spike at time *t*, the probability that it was elicited in ensemble 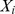 is 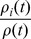, where 
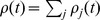
 denotes the total network rate. Since the total network rate 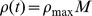 is constant and each spike is drawn independently, to count the spikes in different ensembles in a time window 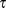, we can replace each inhomogeneous Poisson process by a homogeneous process in that time window with rate 
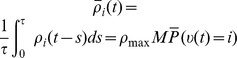
, where 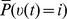 denotes the temporally filtered probability of the state, see eq. (21). Each individual spike in the time window originates from ensemble 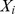 with probability 
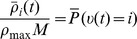
. Hence, for a given 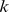, 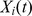 is drawn from a binomial distribution 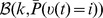. It follows that 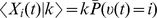 and 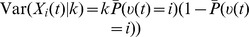. Hence, 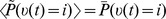, and the estimator is unbiased.

We now turn to the variance of 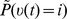. We define 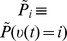 and 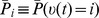 for notational convenience. From 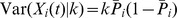, we obtain 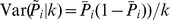. By the law of total variance, we have
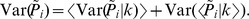



The second summand is 0 since 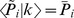 is independent of 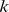. We thus obtain
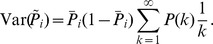



Here, 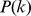 is Poissonian with intensity 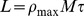. Inserting the Poisson density, we obtain
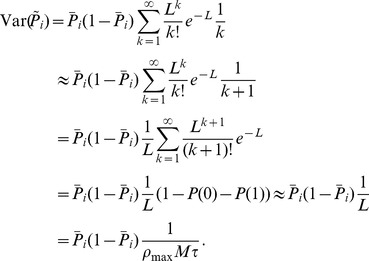



The approximation is excellent if 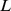 is large, i.e., a small number of spikes over all ensembles within 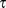 is very unlikely.

### Influence of the firing rate on the represented distribution

We provide here the proof for eq. (2). We assume that an antiderivative 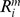 exists for all rates 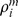 (which is satisfied for example if the rates are continuous or sums of Heaviside step functions). The probability mass of a population 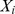 is given by
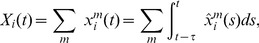
(23)where 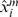 is the spike train of neuron 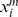 as defined above. The neurons spike in a Poissonian manner with continuous rates 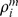. Hence we obtain for the mean over realizations of spike trains
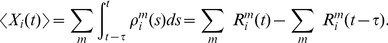
(24)It follows
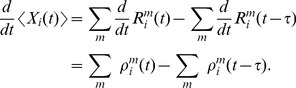
(25)


### Task class A: Simple probabilistic dependencies

We first show that the expected value of the estimator 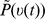 is equal to the posterior distribution (4) for membrane potentials (5) and weights (6). We will then derive the variance of an alternative estimator 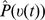 of the posterior distribution.

Consider two distributions 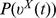 and 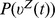 over random variables 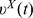 and 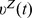 respectively such that the desired posterior is given by eq. (4). 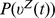 is coded by neurons 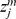 through ENS coding. We denote the estimation sample size of the ensembles for 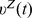 by 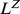 for clarity. Consider a circuit with neurons 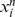 and ensemble size 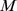 that should represent the posterior. The membrane potentials are given by 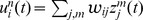 for 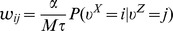. For given EPSP-filtered spike trains 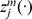, this leads to the firing rate for neuron 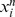


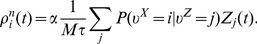
(26)


Averaging over realizations of spike trains in the posterior population, we obtain
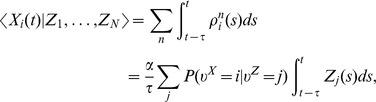
where 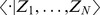 denotes the average for given activities in ensembles 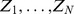. Taking also the average over realizations of spike trains in ensembles 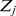, this evaluates to



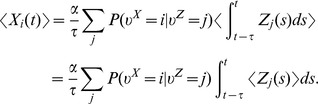



We assume for simplicity that 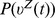 is constant over time, and obtain
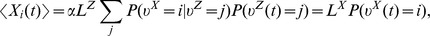
where we defined 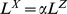. This shows that the represented probability of the circuit is the posterior probability in the mean with an estimation sample size of 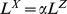.

#### Variance of the posterior representation

The estimate of the posterior distribution at some specific time *t* is however variable due to variability in spike counts of both the representation of 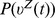 and the representation of 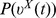. Due to this doubly stochastic nature, the variance of the estimator 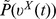 is hard to evaluate. We derive in here the variance of an alternative estimator 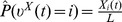 which is also unbiased but has higher variance. We show that the variance of this estimator for conditional probabilities 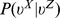 that maximize the variance of the firing rates in the posterior circuit is at most twice the variance of the estimator 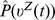. The firing rate of a neuron in the posterior representation has a variance of
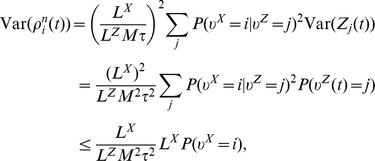
where the maximum is achieved when one 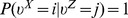 for some *j*. We derive the variance of the spike count in a window of size 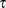 in the posterior circuit in this case. This variance is not straight forward to compute since it is the variance in spike count of a Poisson process with a rate that is itself a random variable. For the case of maximum variance of the firing rate, the firing rate is given by
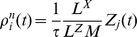
for some *j*. For the spike count in a window of duration 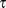 over the whole ensemble for state 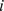, that is 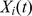, we consider thus a Poisson-distributed random variable with intensity 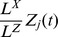 which is itself a random variable. Since 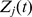 is Poisson distributed, 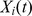 can be expressed as a compound Poisson distribution in the following way. 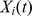 is the sum of random variables 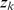 which are i.i.d. Poisson with intensity 1 and scaled by 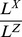. The number of random variables that are summed is Poisson distributed with intensity 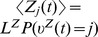. Hence, the random variable 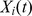 is given by
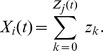
It is easy to see that this leads to the correct distribution over intensities for 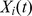. It is known from the theory of compound Poisson processes [51] that the variance of 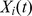 is given by 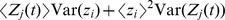 which is in our case 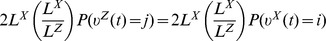. Since the estimated probability for state *i* is 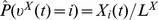, we obtain 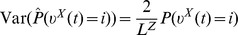. In comparison with the variance of 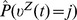, which is 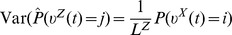, the variance doubles.

### Task class B: Evidence integration

#### Evidence can be provided through EPSPs

We first prove that in a rate-model, integration of evidence signaled via point-events can be performed with finite-length EPSPs. We denote the spike-train 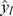 of afferent neuron 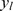 as the sum of Dirac delta functions
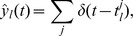
where 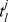 denotes the *j*-th spike-time of neuron 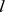. Consider a set of non-normalized and non-negative functions 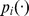 from which the probabilities 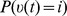 follow after normalization. The change of 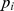 is given by [13], [14]



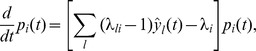
where 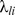 is the firing rate of afferent neuron 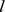 if the current state is 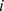. Starting at some time 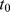 with 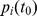 and integrating up to time 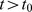, we obtain
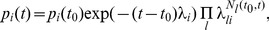
(27)where 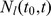 denotes the number of spikes of afferent neuron 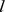 in 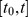.

Now assume finite-size EPSPs of length 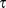 and integral 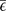, giving rise to the EPSP-filtered spike-trains 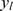. Furthermore, assume that the rate-based network evolves due to eq. (8) with weights given by 
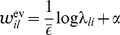
 for some constant 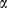. Integrating up to time 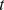 and assuming no evidence spike in 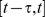, we obtain
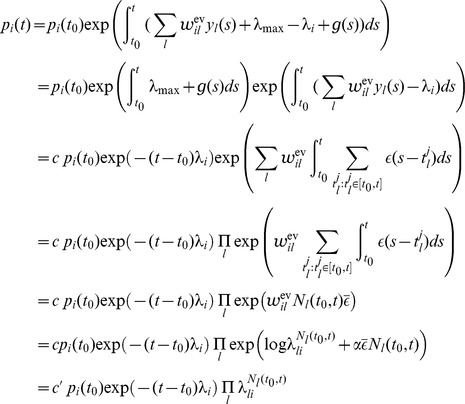
for a constant 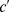 that only scales the 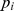
's. We thus obtained the desired result, compare to eq. (27). Note that 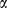 can be used to shift weights to positive values for low firing rates. Since the EPSPs are scaled by the weight, we can assume that 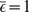. Then the optimal weights are 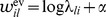, as considered in the main text.

#### Particle-based implementation of the filtering equations

We now analyze the changes of expected probability masses for membrane voltages given by eq. (10) with 
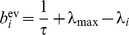
 and 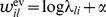. We show that they are approximately equal to the changes in the 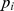
's in eq. (8). For a given pattern of spikes in layer 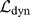, the firing rates in layer 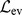 evaluate to
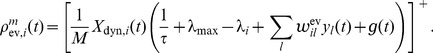
(28)


For positive weights, the only term that can make the argument of the 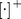 operator negative is 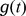 which will be equated with normalizing inhibition. We will discuss the influence of this effect below. For now, we assume that the membrane potential does not become negative, which enables us to skip the rectification 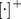. We use the shortcut 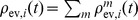 for the summed firing rate of ensemble 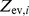 to obtain
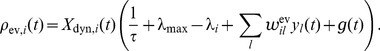
(29)


The change of the expected probability masses in 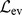 is given by eq. (2)
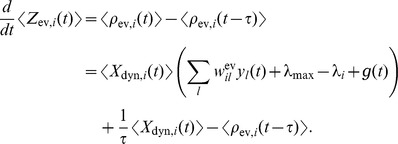



Since layer 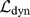 copies the distribution represented by 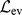, we have 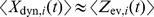 which yields
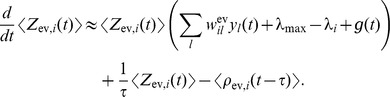



The first term in this equation is equivalent to the change of the 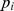
's in eq. (8). The last term is due to EPSPs that end at time 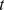 and has to be compensated. It is approximately compensated by the second to last term since 
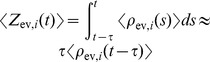
 under the assumption that the expected firing rate (i.e., the represented probability distribution) changes slowly on the time scale of the EPSP. Hence, we have
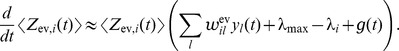



Comparing this result to eq. (8), we can see that the dynamics of the mean probability masses in layer 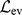 approximate those needed for evidence integration.

#### Multiplication through gating of activity

We now show that the multiplication can be approximated by gating of activity. The membrane potentials of neurons in 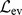 given in eq. (10) gives rise to the ensemble firing rates given in eq. (29), which approximate the optimal changes in probability masses as shown above. In a first step, we show that membrane potentials (11) give rise to identical ensemble firing rates. This can easily be seen since for non-negative membrane potentials we have
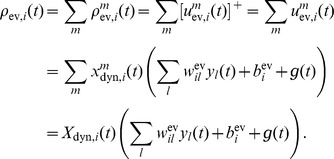



The matching of superscripts *m* in eq. (11) is chosen for notational simplicity. Of course, any permutation of superscripts on the right hand side is valid as well. If the firing rate of neuron 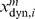 is low, the probability of two spikes in a time window of size 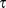 is small and we can approximate 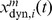 by a binary variable taking on the values 0 or 1. In this case, the multiplication is accomplished by gating of the activity of neuron 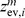 by neuron 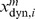.

In a second step, we discuss how this gating can be accomplished through disinhibition. Our general model for disinhibition is discussed below. Here we use the special case of a single disinhibiting neuron 
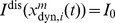
 if 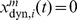 and 0 otherwise. For membrane potentials given by eq. (12), and binary 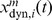, we have
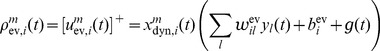
(30)if 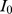 is strong, such that the membrane potential becomes negative if neuron 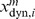 was not active recently. The ensemble rates
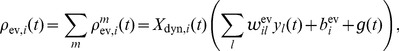
(31)are equivalent to the ensemble firing rates of eq. (29) and therefore approximate the optimal changes of probability masses.

### Disinhibition

We formally define the influence of disinhibition arising from neurons 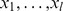 on the membrane potential of a neuron as
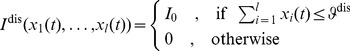
(32)where 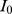 is some baseline inhibition and 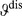 is some threshold. In other words, the disinhibited neuron is released from baseline inhibition if the neurons were recently active enough to overcome the threshold 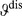. We used in this article a threshold of 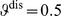. For rectangular EPSPs, this results in disinhibition whenever 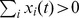. In our circuit model, the baseline inhibition is strong enough to suppress any activity when inhibited.

### Lateral inhibition

Lateral inhibition in layer 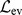 is given by
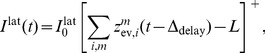
(33)


It corrects for excessive spiking activity, i.e., when 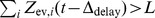. The constant 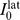 determines how strongly this correction influences the membrane potential. We included the 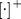 operator to model a purely inhibitory population. This means that too low firing rates are not compensated by this mechanism. This is possible, since all other contributions to the membrane potential are non-negative if weights are shifted to non-negative values. In particular, the 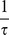 term in the biases 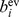 (see Table 1) leads to a quick recovery of the ensemble firing rate.

Strong lateral inhibition at low potentials could be cut off by the 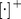-operator in equation (28). That would result in unequal inhibition values for different ensembles. This can be avoided by bounding inhibition to a maximum of 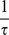 (note that inhibition is bounded due to the finite size of the network). But our simulations indicate that even with larger values, the circuit performs very well.

### General details on computer simulations

All simulations were performed in discretized time with a time step of 0.5 ms. The duration of EPSPs was set to 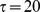
ms in all simulations. The delay for lateral inhibition 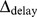 was set to 0.5 ms. The initialization of network activity for the particle filter circuits was performed as follows. Let 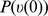 denote the distribution that the network should represent at the beginning of a simulation. Spikes were drawn in both layers and distributed in the time interval 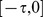 such that at time 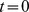, the represented probability 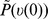 represented 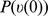 in the mean. More precisely, a spike of neuron 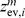 in 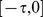 was assumed with probability 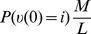. If there was a spike for this neuron, its exact timing was drawn from a uniform distribution in the interval 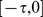. Simulation of the network began at *t* = 0 ms where the EPSPs of theses spikes were taken into account when computing the membrane potentials of neurons during the initial phase of the simulation. The initially represented distribution 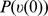 was assumed uniform over all states if not otherwise noted.

All simulation code is available online as *Dataset S1*. Details of individual simulations are discussed in the following.

### Computer Simulations for task class B: Evidence integration

#### Figure 5

For panel F, the prior probability for state 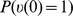 was chosen in each run randomly from a uniform distribution over 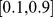. The two afferent neurons 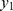 and 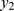 spiked at time 20 ms and 25 ms respectively. Each 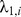 was drawn from a uniform distribution over [5], [20]Hz. For panel F, 100 simulation runs were performed and 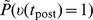 as well as 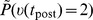 is shown, with 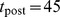
ms (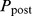). The true posterior 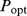 was computed according to eq. (27). Panels C–E show an example run with 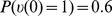 and 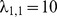
Hz, 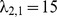
Hz, 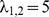
Hz, 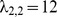
Hz.

Circuit parameters: Number of neurons per state 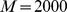; target estimation sample size 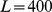; lateral inhibition scaling 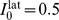.

### Action readouts from state variables

We provide here details on the readout layer used to model the ambiguous target task and the random-dot motion task. An application of the feedforward circuit discussed in *Simple probabilistic dependencies* is inference over rewarded actions 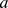 based on the current belief about the state of a random variable 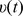. In the context of reward-based action selection, one wants to estimate the probability that a given action leads to reward (we consider here binary rewards 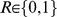 for simplicity). If the probability 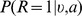 of reward for action 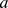 in state 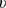 is known, then the posterior distribution over actions for the current state distribution can be inferred as
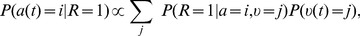
(34)see below. In the ambiguous target task, 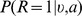 is 1 if the action (movement direction) matches the direction of the spatial cue in the color of the color cue, and 0 otherwise. Eq. (34) defines a direct probabilistic relation between the action and the state. Hence, a layer of neurons as described in *Task class A* computes the posterior distribution. We refer to such a layer as an *action readout layer*.

We discuss this now more formally. Consider a random variable 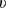 with range 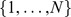, a random variable 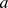 over possible actions 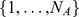, and a binary random variable 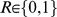 that indicates whether a reward occurs. Assuming that the joint probability 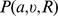 factorizes to 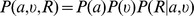, one can infer future actions 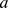 that will lead to a reward for the given distribution over states by:
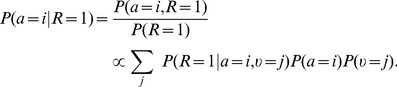
(35)


We want to infer future actions 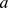 that will lead to a reward for the current state 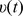. Assuming a uniform prior over actions, this simplifies to
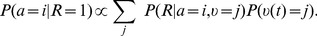
(36)


Assume that the distribution 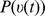 is given by ensembles 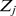 in a sample-based representation with estimation sample size 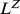. This defines an operation as discussed above under *Task class A: Simple probabilistic dependencies*. Hence, a circuit consisting of 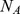 ensembles and 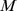 neurons per ensemble samples from the posterior distribution (36) with estimation sample size 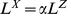 if the membrane voltages of the neurons are given by
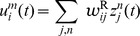
with weights 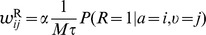.

### The ambiguous target task

Stimuli were coded by 19 ensembles of afferent neurons, where each ensemble consisted of 20 neurons. One ensemble coded for the fixation cross, and two for the two colors of the color cue. Eight ensembles coded for each position of a red spatial cue and eight for each position of a blue color cue. First, the fixation cross-stimulus was presented for 100 ms. The spatial cue followed for 250 ms. Here, two ensembles of afferent neurons were active, one for each color of the spatial cue. In the 250 ms memory epoch, only the fixation cross was present. After the memory epoch, the color cue appeared, and the corresponding ensemble of afferent neurons (for red or blue color cue) was active. Afferent neurons spiked at a baseline rate of 0.1 Hz and at a rate of 5 Hz when they were active.

For synaptic weights from afferent neurons, we used 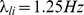 for observations 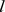 that are possible in state 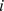. For example, in a state coding for “movement to 90 degrees with red color cue”, a red spatial cue at 90 degrees, a blue spatial cue at 270 degrees, and a fixation cross are possible. All other 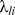 were set to the baseline firing rate of 0.1 Hz. The lateral inhibition scaling was 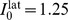.

The action readout layer consisted of 100 neurons for each of the 8 possible actions (movement to one of eight directions). Considering eq. (4), we identified the random variable 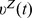 with the random variable estimated in the particle filter circuit. The hidden states of this variable are the direction-color pairs 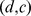 for 8 possible directions 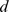 and 2 colors 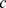. The random variable 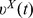 has 8 states encoding that movement in direction 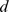 leads to a reward. This random variable can be computed through marginalization over color 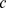. Hence, the conditional probabilities were given by 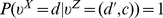 for 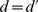 and 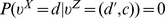 otherwise. With these conditionals, we obtained the synaptic weights through eq. (6) with 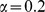, leading to an estimation sample size of 80 in the action readout layer.

For Figure 1, spiking activity was averaged over 50 successful trials (87 successful trials out of 100), temporally (10 ms running average) and spatially (mean activity of 10 neighboring neurons for neurons ordered by their preferred direction).

### Continuous time Markov chains

A continuous time Markov chain is characterized by *transition rates* 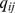 for each pair of distinct states, i.e., for 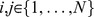 and 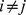. Assume that at some time 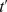 the state is 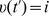. To sample a sequence of states from the Markov chain starting at 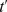, a Poisson process 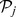 with rate 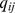 is started for each state 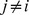. The time of the next state transition is given by the first event in these processes and the next state is given by the index of the process that produced the event. After a transition, the chain starts afresh at the transition time.

### Task class C: Bayesian filtering

We show that the membrane potentials given in Table 1 approximate the set of differential equations (13). These equations can be formulated as two sets of coupled equations, one for evidence integration and one for prediction of dynamics:
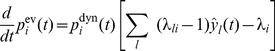
(37)

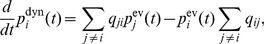
(38)where we assume identical initial conditions for 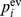 and 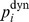. 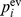 is represented by 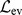 and the change of the expected probability masses corresponds to the changes in eq. (37) as shown above.

We show here that the membrane potentials given in eq. (14) lead to changes in the expected probability masses of 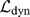 that approximate those of eq. (38). Assume that the membrane potentials evolve according to eq. (14) with weights 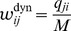 where we defined 
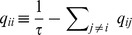
. For given spikes in layer 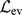, the summed firing rates 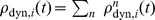 in layer 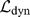 evaluate to
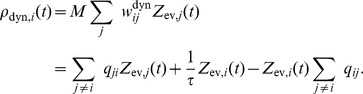



The change of the expected probability masses in 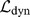 is
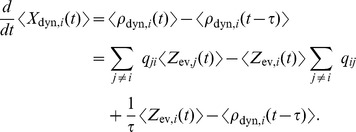



The last term is due to EPSPs that end at time *t* which needs to be compensated. It is approximately compensated by the second to last term since 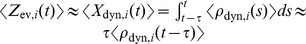 under the assumption that the expected firing rate (i.e., the represented probability distribution) changes slowly on the time scale of the EPSP. Altogether we obtain
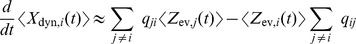
as needed.

### Computer Simulations for task class C: Bayesian filtering

#### Figure 7

Parameters for the example run, panels C,D: 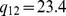
Hz; 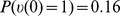. The true posterior was computed analytically as
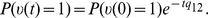



Circuit parameters were chosen identical to those for Figure 5: Number of neurons per state 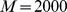; target estimation sample size 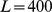; lateral inhibition scaling 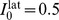.

#### Particle filtering in a generic setup for task class C

State sequences were generated according to the HMM described in the main text. Once a new state was drawn, it was maintained for at least 60 ms. Point-event observations were produced with rates
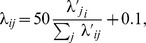
where 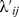 were set according to Gaussian tuning functions with means 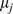 and standard deviation 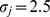 for all *j*



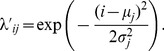
(39)The means were set to 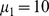, 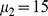, 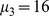, 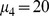, 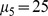. Weights from evidence neurons to the evidence layer were set according to these observation rates.

Circuit parameters: Number of neurons per state 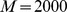; target estimation sample size 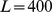; lateral inhibition scaling 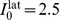. The circuit was initialized at 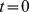 with the prior distribution 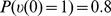 and 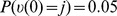 for 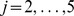.

Panel Af: All simulations were performed in discrete time with a discretization time step of 0.5 ms. The optimal Bayesian filtering was performed by implementing the filtering equations (37) – (38). The optimal estimate based on the most recent observation was computed in the following way: If there was no observation at time 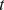, the estimate was identical to the estimate at time 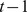. If there was an observation 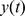 at time 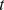, then the distribution over states was given by the posterior 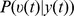. The errors were computed as follows: At each discrete time step, the maximum over the posterior distribution was taken as the predicted state 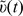. The error was then computed as the fraction of incorrect predicted states 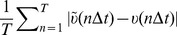, where 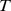 is the number of time steps of the simulation run and 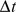 is the discretization time step.

### Computer Simulations for task class D: Context-dependent Bayesian filtering

#### Particle filtering in a generic setup for task class D

State sequences were generated according to the HMM described in the main text, where state transition rates were chosen according to the current context. The HMM started in context A and context was switched whenever state 1 was entered. Point-event observations were produced as in *Particle filtering in a generic setup for task class C*, but with different tuning functions. The tuning functions for states 1 to 3 were Gaussians with 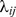 defined as in eq. (39) with means 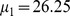, 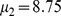, 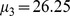 and standard deviations 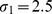, 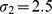, 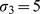. Note that the tuning curves for states 1 and 3 differed only in their standard deviations, but not in their means. The tuning for state 4 was given by a sum of two Gaussians with 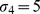 with an additive offset
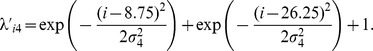



The tuning for state 5 was uniform 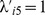. Weights from evidence neurons to the evidence layer were set according to these observation rates.

Circuit parameters were identical to those in *Bayesian filtering in a generic setup*. 10 context neurons were used per context. They produced Poisson spike trains with rate 50 Hz when active and no spikes otherwise.

Panel Bf: Simulations were performed and errors were computed as described in *Particle filtering in a generic setup for task class C*. For the optimal context-dependent Bayesian filtering (“opt”), the optimal transition rates were used for the current context. For the optimal filtering without context (“mix”), the weights to layer 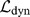 were set according to the mixed transition rates 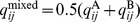, where 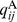 and 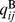 are the transition rates for context A and B respectively.

#### Self-localization

For the full maze, we used coordinates between 0 and 1 in each dimension. Locations of variables were uniformly spaced on a 10×10 grid in the maze (see Figure 10A). We simulated an agent that navigated in the continuous space. Let 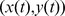 denote the location of the agent at time 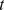. All afferent neurons had a baseline firing rate of 0.1 Hz. The firing rate of each afferent neuron was given by a Gaussian with a corresponding center, an STD of 0.1, and a maximum rate of 50 Hz. The lower four afferent neurons in Figure 10A, had identical positional tuning in both chambers. Their firing rate at each time 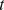 was determined by the Gaussian in the chamber where the agent was currently situated. For 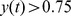, all afferent neurons spiked at baseline.

Circuit parameters: The network size 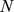 and estimation sample size 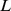 was 250; 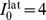. 10 context neurons were used per context ensemble, each producing a 50 Hz Poisson spike train in its context and no spikes if the context did not match. The weights from evidence neurons to neurons in 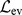 were set according to the rates of the afferent neurons when the agent would be exactly at the place of the corresponding state. The weights to neurons in layer 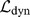 were set according to transition rates as follows. There were four groups of populations in this layer, one for each context. Transition rates were 3.5 Hz for possible movements in the direction that corresponded to the context of the population (dark gray arrows in Figure 10A). Transition rates between other states with adjacent positions (horizontally, vertically, or diagonal; states separated by the chamber wall were considered non-adjacent) were 0.1 Hz (light gray lines in Figure 10A). Transition rates between remaining states were 0 Hz.

### Task class E: Graded integration of uncertain context information

In task class E, it is advantageous to deal with context in a graded manner. This is achieved by a simple modification the context-dependent filtering circuit from task class D. In the modified circuit, the membrane potential of neurons in 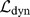 is given by
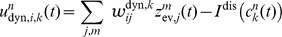
where 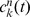 denotes the EPSP-filtered spike train of the 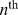 neuron in the 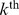 ensemble that represents the distribution over contexts. In comparison to the equation given in Table 2, each neuron in 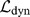 is disinhibited by a single context neuron. This leads to an approximate linear mixture of the context-dependent transition rates



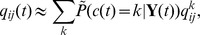
where 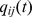 is the effective transition rate for the context-dependent filtering circuit at time 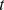 and 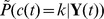 is the estimated posterior for the context variable at time 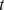.

### Computer simulations for task class E: The ambiguous target task revisited

State sequences and spike trains of evidence neurons were generated as in the simulation for the ambiguous target task, with the exception that the fixation phase lasted for 250 ms. Circuit parameters for the context-dependent filtering of 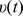 and the action readout layer were identical to those in the simulation for the ambiguous target task (1000 neurons per sate; estimation sample size 400; 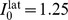). In the fixation-context, transition rates between all pairs of states were 1 Hz.

A particle filter circuit (without context) was employed to estimate 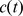, the current phase of the trial. The basic parameters for this circuit were identical to the parameters of the context-dependent filtering circuit (1000 neurons per sate; estimation sample size 400; 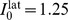). Other parameters for this circuit were as follows. Synaptic weights to 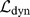 were set according to transition rates that were assumed to be 5 Hz between states where a transition is possible and 0 Hz between other states. Synaptic weights from evidence neurons to the evidence layer were set according to the following observation rates (see also Figure 11). A fixation cross in the CHT state or in the MEM state: 5 Hz; Any spatial cue in the SC state: 5/16 Hz; Any color cue in the CC state: 2.5 Hz; For other observation rates, a baseline of 0.1 Hz was assumed.

## Supporting Information

Dataset S1 **Matlab source files for all simulations.**
(ZIP)

Text S1 **Interpretation of EPSPs as the validity of a spike as a sample.**
(PDF)

Text S2 **Cue combination in ENS coding.**
(PDF)

Text S3 **Value-related neuronal activity through particle filtering in ENS coding.**
(PDF)

Text S4 **Features of neuronal activity during random-dot motion tasks.**
(PDF)

Text S5 **Probabilistic reasoning in area LIP.**
(PDF)
