# Supplementary figures and images for: Ensembles of Spiking Neurons with Noise Support Optimal Probabilistic Inference in a Dynamically Changing Environment
Source: PLoS Comput Biol. 2014 Oct 23;10(10):e1003859. doi: 10.1371/journal.pcbi.1003859 (PMC4207607; doi:10.1371/journal.pcbi.1003859)

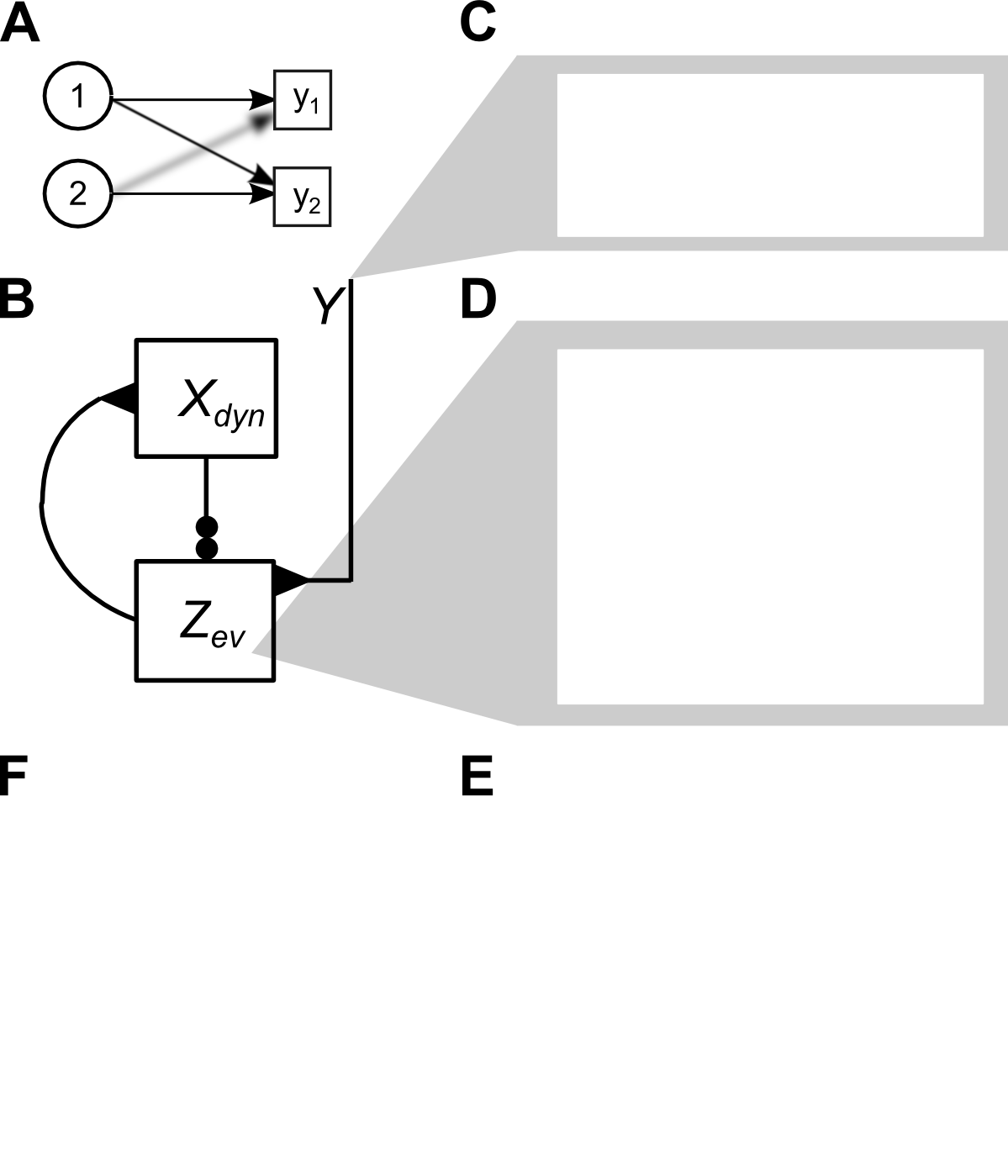

Supplement: Dataset S1 — Matlab source files for all simulations. (ZIP) [file pcbi.1003859.s001.zip › SuppData_S1/fig05_evidence_test/circuit_schema_ev_test.png]

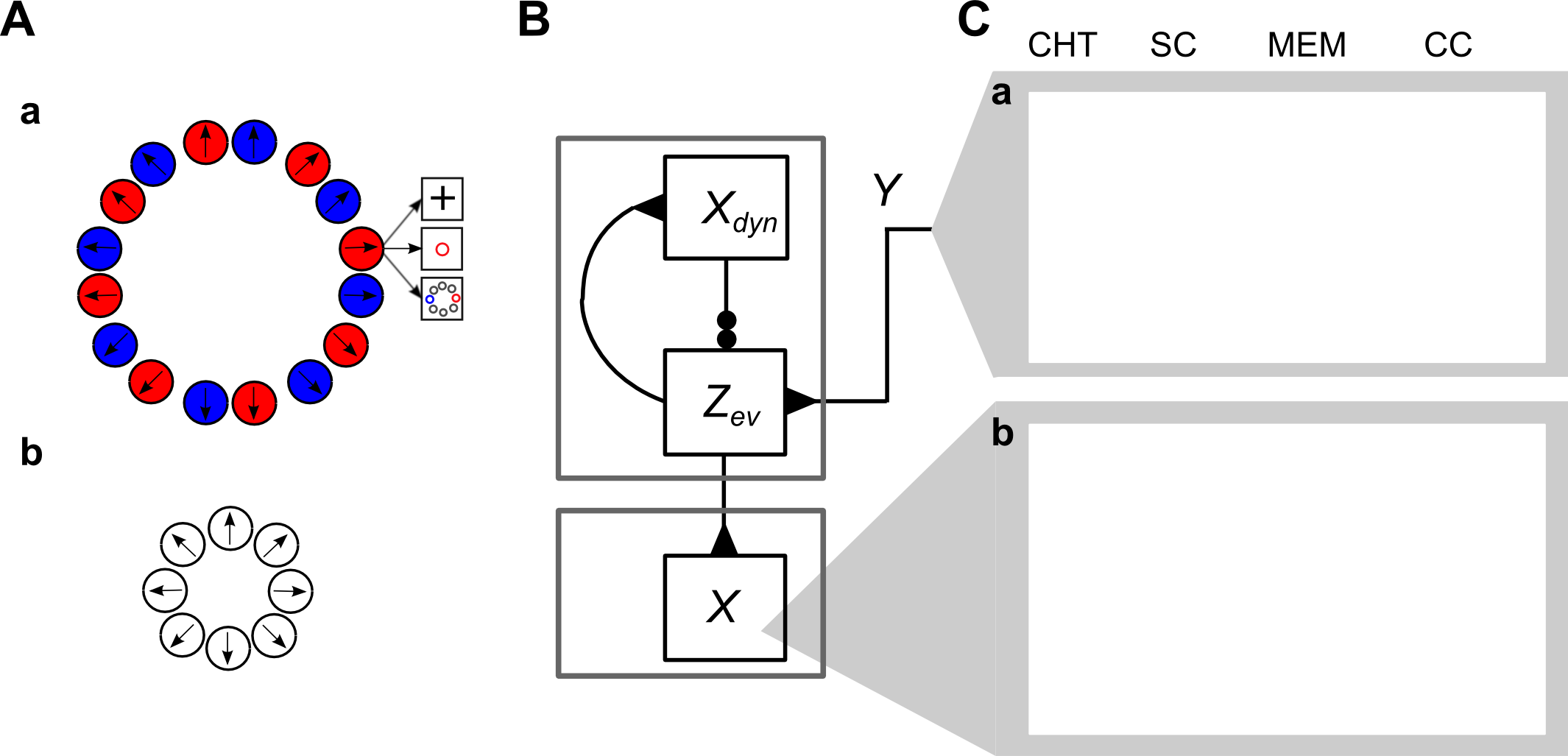

Supplement: Dataset S1 — Matlab source files for all simulations. (ZIP) [file pcbi.1003859.s001.zip › SuppData_S1/fig06_cisek/circuit_schema_cisek.png]

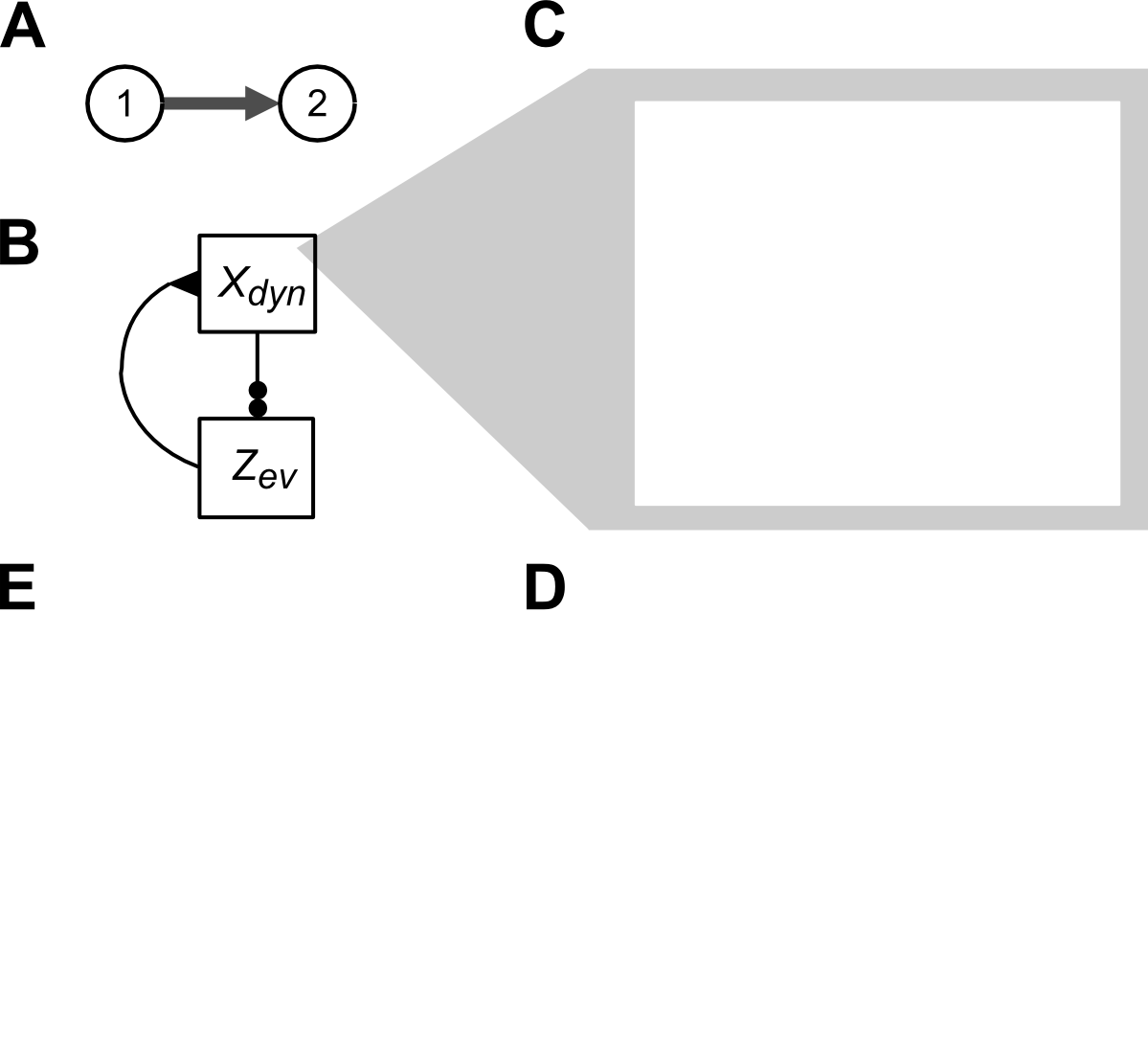

Supplement: Dataset S1 — Matlab source files for all simulations. (ZIP) [file pcbi.1003859.s001.zip › SuppData_S1/fig07_pr_test/circuit_schema_pr_test2.png]

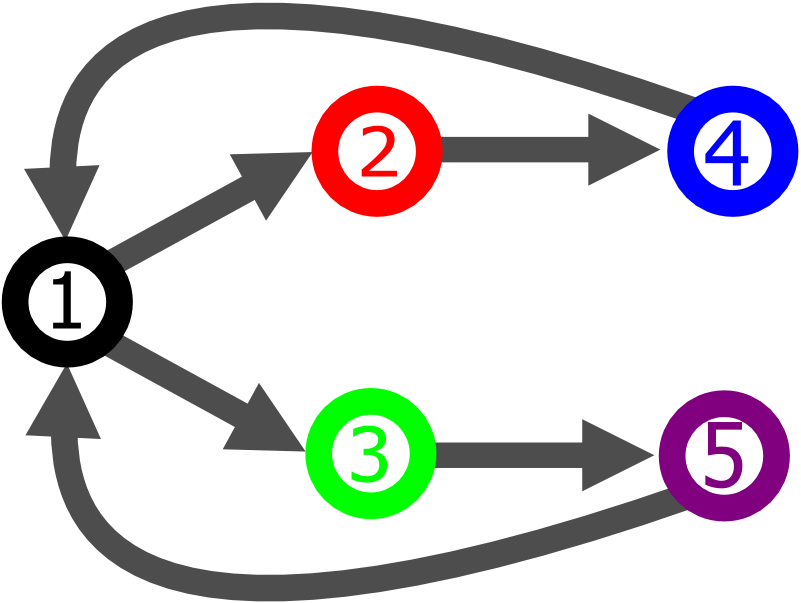

Supplement: Dataset S1 — Matlab source files for all simulations. (ZIP) [file pcbi.1003859.s001.zip › SuppData_S1/fig08_bf/hmm_fork3.png]

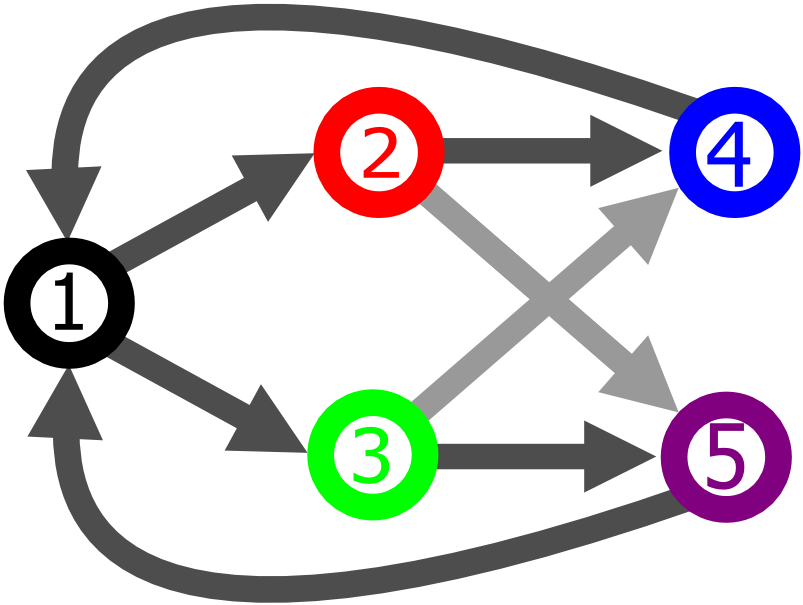

Supplement: Dataset S1 — Matlab source files for all simulations. (ZIP) [file pcbi.1003859.s001.zip › SuppData_S1/fig08_bf/hmm_fork_context3.png]

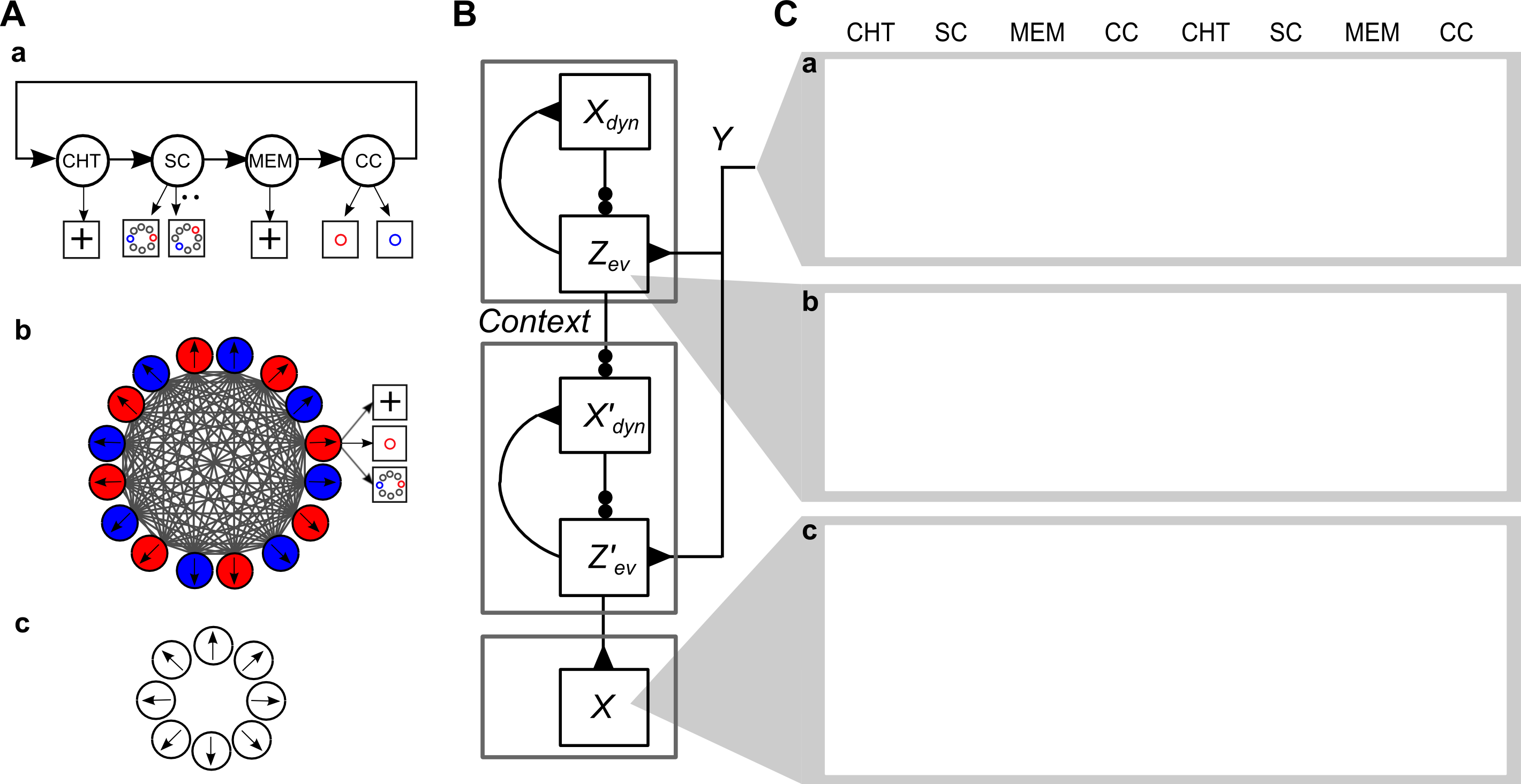

Supplement: Dataset S1 — Matlab source files for all simulations. (ZIP) [file pcbi.1003859.s001.zip › SuppData_S1/fig11_cisek_context/circuit_schema_cisek_context.png]
