# Supplementary material for: Ensembles of Spiking Neurons with Noise Support Optimal Probabilistic Inference in a Dynamically Changing Environment
Source: PLoS Comput Biol. 2014 Oct 23;10(10):e1003859. doi: 10.1371/journal.pcbi.1003859 (PMC4207607; doi:10.1371/journal.pcbi.1003859)
Supplement: Text S1 — Interpretation of EPSPs as the validity of a spike as a sample. (PDF) [file pcbi.1003859.s002.pdf]

Supporting Text S1 for: Ensembles of spiking neurons with noise support optimal probabilistic inference in a dynamically changing environment

Robert Legenstein*, Wolfgang Maass,
Institute for Theoretical Computer Science
Graz University of Technology
A-8010 Graz, Austria
∗ **E-mail:** robert.legenstein@igi.tugraz.at

Interpretation of EPSPs as the validity of a spike as a sample

We have assumed that downstream neurons decode a represented distribution through the integration of spikes over a constant time window given by the EPSP length τ with rectangular EPSPs. In this time window, an average of L spikes is emitted by the network. A motivation for rectangular EPSPs can be derived by assuming that the goal of the decoding neurons is to integrate a number of samples that is is equal to L. EPSPs can then function as a proxy for the *validity* of the sample, that is, the probability that it belongs to the set of the L most recently emitted ones. The temporal shape of this validity (and therefore the shape of the optimal EPSP) depends on the estimation sample size L. For $L = 1$, the optimal EPSP shape is an exponentially decaying function with time constant τ. It becomes more plateau-like for increasing L and approaches a rectangular shape of width τ for large L (see Fig. 1 and the derivation below). For large L, the optimal shape thus approaches the rectangular shape used in this article. A common feature of all these shapes is that the expected time until a spike becomes invalid as a sample is τ.

We derive in the following the probability that a spike at time t belongs to the set of the L most recently emitted ones at time $t + \Delta t$. An optimal EPSP would weight spikes according to this validity. Consider an ensemble of M neurons with estimation sample size L, i.e., the total firing rate of the circuit is $\frac{L}{\tau}$. We say that a spike elicited at time t by some of these neurons is a valid sample at time $t + \Delta t$ if it belongs to the last L spikes up to $t + \Delta t$, i.e., if the number of spikes $N_{t,\Delta t}$ in $(t, t + \Delta t]$ is smaller than L. Assuming that spikes are emitted according to a

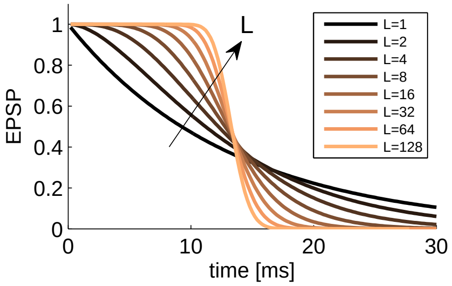

Figure 1. Validity of a spike as a sample. Shown is the probability that a spike at time $t = 0$ belongs to the last L spike-events when a set of neurons fire according to a Poisson process with total rate L/τ with $\tau = 13$ms. Varying values of L are indicated by color with increasing L along the arrow.

Poisson process, the distribution over $N_{t,\Delta t}$ is

$$P(N_{t,\Delta t} = l) = \mathrm{Poiss}(l; \frac{L}{\tau}) = \frac{(L\Delta t/\tau)^l}{l!} \exp(-\Delta t/\tau), \tag{1}$$

where $\mathrm{Poiss}(l; \lambda)$ denotes the Poisson distribution over l with rate λ. Hence, we have

$$P(N_{t,\Delta t} < L) = \sum_{l=0}^{L-1} \mathrm{Poiss}(l; \frac{L}{\tau}). \tag{2}$$

For $L = 1$, this results in an exponential decay with time constant τ. For larger L, the shape becomes more plateau-like.
