# Supplementary material for: Ensembles of Spiking Neurons with Noise Support Optimal Probabilistic Inference in a Dynamically Changing Environment
Source: PLoS Comput Biol. 2014 Oct 23;10(10):e1003859. doi: 10.1371/journal.pcbi.1003859 (PMC4207607; doi:10.1371/journal.pcbi.1003859)
Supplement: Text S2 — Cue combination in ENS coding. (PDF) [file pcbi.1003859.s003.pdf]

Supporting Text S2 for: Ensembles of spiking neurons with noise support optimal probabilistic inference in a dynamically changing environment

Robert Legenstein*, Wolfgang Maass,
Institute for Theoretical Computer Science
Graz University of Technology
A-8010 Graz, Austria
$\ast$ **E-mail:** `robert.legenstein@igi.tugraz.at`

Cue combination in ENS coding

Cue combination, as considered for example in [1], is a nonlinear operation in ENS coding. In cue combination, evidence about a random variable $v(t)$ is given in terms of the activity of two ensembles of neurons x_i^m and y_j^n coding two distributions $Q^X(v(t))$ and $Q^Y(v(t))$. The activities of these two ensembles may result for example from the visual and auditory modality respectively. The task is to combine this evidence in a posterior distribution $P(v(t))$ represented by a third ensemble z_k^l. If the activities in x_i^m and y_j^n are independent given the stimulus, then it is optimal to combine the distributions multiplicatively such that the desired posterior is given by $P(v(t)=i) \propto Q^X(v(t)=i)Q^Y(v(t)=i)$. If x_i^m and y_j^n represent the distributions through ENS coding, then a circuit with membrane potentials

$$u_i^m(t) = \frac{1}{L}\sum_n y_i^n(t) - I^{\mathrm{dis}}(x_i^m(t)) - I^{\mathrm{lat}}(t) \tag{1}$$

combines the cues and z_k^l represents the approximate posterior in ENS coding. Again, the multiplication is approximated by disinhibition.

References

1. Ma WJ, Beck JM, Latham PE, Pouget A (2006) Bayesian inference with probabilistic population codes. Nat Neurosci 9: 1432–1438.
