# Supplementary material for: Ensembles of Spiking Neurons with Noise Support Optimal Probabilistic Inference in a Dynamically Changing Environment
Source: PLoS Comput Biol. 2014 Oct 23;10(10):e1003859. doi: 10.1371/journal.pcbi.1003859 (PMC4207607; doi:10.1371/journal.pcbi.1003859)
Supplement: Text S3 — Value-related neuronal activity through particle filtering in ENS coding. (PDF) [file pcbi.1003859.s004.pdf]

Supporting Text S3 for: Ensembles of spiking neurons with noise support optimal probabilistic inference in a dynamically changing environment

Robert Legenstein*, Wolfgang Maass,
Institute for Theoretical Computer Science
Graz University of Technology
A-8010 Graz, Austria
∗ **E-mail:** robert.legenstein@igi.tugraz.at

Value-related neuronal activity through particle filtering in ENS coding

Action-predictive activity in macaque motor cortex is also modulated by the expected value of the action. This was demonstrated in [1]. There, experiments were performed where a visual cue indicated the value of an action in a planar center-out-reaching task. The task was similar to the task of Cisek and Kalaska (2005) in the sense that a visual cue indicated two of eight possible target directions for a subsequent movement. It differed in three respects. First, there was no color cue. Instead, after the visual cue, a go-cue appeared and the movement had to be performed immediately. Second, any possible direction pair could occur instead of the restriction to directions with a 180° offset in the former task. Finally and most crucially, the border style of the cue in each direction indicated probabilistically the number of juice drops for that action. A "low-value" target (disk with thick black border) had a 60% chance of yielding 1 drop, 30% chance of yielding 2 drops, and a 10% chance of yielding 3 drops. For the "medium-value" target (disk without border), the probabilities were 60% for 2 drops, 20% for 1 drop, and 20% for 3 drops. A "high-value" target (disk with thin border) was worth 3 (60%), 2(30%), or 1 drop (10%) of juice. Experiments were performed with a single target (1-target task) and with two concurrent targets (2-target task). After the go cue, the monkey was free to move to the target of his choice (the authors also considered a task where the monkey was forced to move to a particular target by a corresponding cue, but this task is conceptually similar to the free task with one target).

For conceptual simplicity, we redefine the task such that the value (1 to 3 drops of juice) is converted to a probability of receiving a binary reward. The probability of receiving the

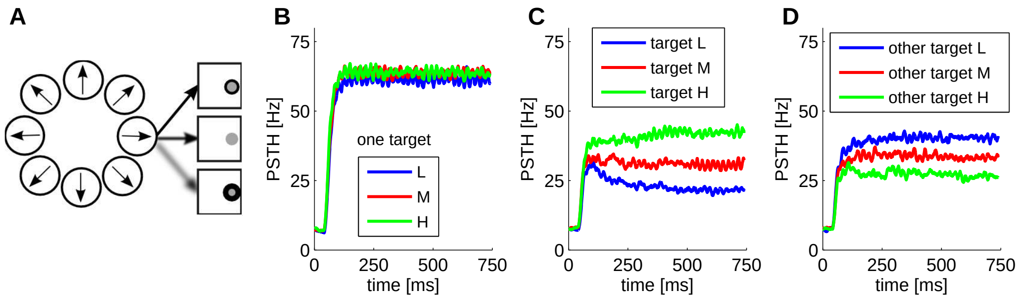

Figure 1. Reward-related firing activity in a reaching task [1]. A) Hidden random variable of the particle filter circuit. For each state of the variable, three observations are expected with rates as indicated by the blurriness of arrows (in decreasing order: circle with thin, without, or thick border at this location). **B-C)** Mean activity of an ensemble of simulated PMd neurons tuned to a given target direction (each data point is the mean over 20ms; 1000 simulations for each panel). **B)** One-target task. Although the target-value is varied to be low (black), medium (red) or high (blue), the neuron activation is constant. **C)** Two target task; The cue in the target direction of the neurons was varied, the other cue was always medium valued. **D)** The cue in the target direction of the neurons was always medium valued. The cue for the other direction was varied. Such modulation of neural responses has been observed in monkey experiments, compare to Fig. 1B-D in [1].

binary reward was either 1 (corresponding to 3 drops of juice), 2/3 (2 drops), or 1/3 (1 drop). Other monotonic relationships lead to qualitatively similar results. For the circuit model, we formalized this task as evidence integration for a hidden random variable with eight states, see Fig. 1A. The hidden variable encoded the rewarded movement action. Observations were the circles with three different border types at each of the eight screen positions, plus an observation for the fixation cross (resulting in 25 observations in total). Each observation was encoded by 20 evidence neurons that produced Poisson spike trains at a rate of 6Hz in the presence of the observation and 0.1Hz otherwise. Synaptic weights from evidence neurons to neurons in $\mathcal{L}_{\text{ev}}$ were set according to hypothesized emission rates that were set according to the probability of reward multiplied by 6Hz for cues and 0.6Hz for the fixation cross. For example, from the state for a movement to the right, a circle with thin border (high-value) at this position had a high emission rate, a circle without border (medium value) had an intermediate emission rate, and a circle with thick border (low-value) had a low emission rate, see Fig. 1A. We simulated a

particle filter circuit with an ensemble size of $M = 200$ and estimation sample size of $L = 200$ ($I_0^{\text{lat}} = 5$). For the first 100ms, the fixation cross was presented. Then, one (in the 1-target task) or two (in the 2-targets task) observations were chosen.

In the simulations of the one-target task, the expected reward did not modulate motor neuron activity, since only evidence for a single movement option was present. In this case, lateral inhibition ensures that the only plausible action is activated strongly (Fig. 1B). The situation changed in the two-targets task. In this case, two actions are plausible according to the observations. Differences in the likelihoods of the observations for the two possible actions biased the competition (see Fig. 1C,D). An observation that indicates a higher likelihood for one hidden state leads to an advantage for the corresponding ensemble in the competition. Thus, its firing rate increased and the firing rate of the competing state decreased. Even when we compared two trials with the identical value for a given action, the firing rate of the ensemble was decreased if the competing action had an increased value. This behavior is consistent with experimental results [1].

References

1. Pastor-Bernier A, Cisek P (2011) Neural correlates of biased competition in premotor cortex. J Neurosci 31: 7083–7088.
