# Supplementary material for: Ensembles of Spiking Neurons with Noise Support Optimal Probabilistic Inference in a Dynamically Changing Environment
Source: PLoS Comput Biol. 2014 Oct 23;10(10):e1003859. doi: 10.1371/journal.pcbi.1003859 (PMC4207607; doi:10.1371/journal.pcbi.1003859)
Supplement: Text S4 — Features of neuronal activity during random-dot motion tasks. (PDF) [file pcbi.1003859.s005.pdf]

Supporting Text S4 for: Ensembles of spiking neurons with noise support optimal probabilistic inference in a dynamically changing environment

Robert Legenstein*, Wolfgang Maass,
Institute for Theoretical Computer Science
Graz University of Technology
A-8010 Graz, Austria
* **E-mail:** robert.legenstein@igi.tugraz.at

Features of neuronal activity during random-dot motion tasks

Random-dot motion tasks have been studied extensively in the context of decision making. In such a task, a monkey typically sees dots that move in random directions. A subset of these dots however moves coherently, the size of which depends on the coherence level of the trial. The monkey has to determine the direction of coherent motion to form a decision which is typically communicated by the monkey through an eye movement to one of two target positions on a screen [1, 2].

For simplicity, we considered here a task where only horizontally moving dots are presented. The dots are presented for a given amount of time, then a binary decision is read out from the neuronal activity. In our model, the hidden state of a random variable $v(t)$ with two hidden states $s_{\text{left}}, s_{\text{right}}$ (dominant leftward or rightward motion respectively) is estimated through particle filtering, see Figure 1A. We simulated the same architecture as in the ambiguous target task (particle filtering circuit and a readout layer, see Figure 6B in *Results*) with 1000 neurons per state and an estimation sample size of 200. The circuit received input from 50 afferent neurons that modeled direction tuned neurons in area MT with different receptive field locations. They were divided into two equally sized groups of neurons selective for leftward and rightward motion respectively. During the presentation of a stimulus, each of the neurons was spiking in a Poissonian manner with a constant rate that depended on the dominant direction of the trial, on its directional tuning, and on the coherence level. The rate f_{dd} of neurons tuned to the dominant direction was chosen between 0.55Hz to 0.9Hz in different simulations (mimicking different coherence levels). Neurons from the non-dominant group had a rate of $(1 - f_{\text{dd}})$Hz.

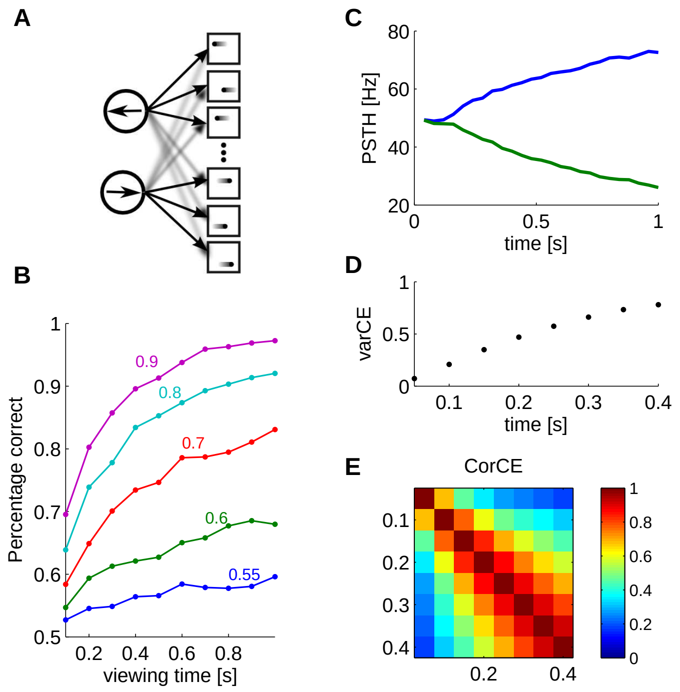

Figure 1. (Legend on next page)

For details on this simulation, see *Methods* in this supporting text.

We measured the percentage of correct decisions conditioned on f_{dd} and presentation time. The "psychophysics" are illustrated in Figure 1B. The model behaves qualitatively similar to monkeys on such tasks, see e.g. Figure 88.2 in [2]. The behavior of single neurons in the action readout is illustrated in Figure 1C. We see integrating behavior as observed in LIP neurons in several studies.

Such integrating behavior can be explained by a number of different models. In order to provide further model constraints, Churchland et al. [3] have analyzed the variability of neuronal activity of LIP neurons in this task. In particular, the authors extracted from the spike counts the variance of the underlying rate itself (as opposed from the variance of the spike generation process), termed the variance of conditional expectation (varCE). The nature of the integrating mechanism determines the temporal evolution of the varCE. In order to test whether our model is consistent with this data, we analyzed the varCE during stimulus integration for a given

Figure 1. Simulated neuronal network behavior in a random-dot task. A) Random variable (circles: hidden states) for which the value is estimated through particle filtering and observation model (squares: observables; blurry arrows indicate observations of low probability). The two hidden states represent dominant leftward and rightward motion respectively. See Figure 6B in *Results* for the circuit architecture. **B)** Percentage of correct decisions for different f_{dd} (indicated by color; related to motion coherence) and viewing time steps in our model (average over 4000 trials per datapoint). Compare to Figure 88.2 in [2]. **C)** Peri-stimulus time histogram for one circuit neuron that codes for the correct decision (blue trace) and one neuron that codes for the incorrect decision (green trace). Shown is the mean activity over time for 6000 trials with $f_{\mathrm{dd}} = 0.75\mathrm{Hz}$ (activity averaged over 40ms bins). **D)** Variance of conditional expectation (varCE) during decision formation. The variance rises approximately linearly over the first 400ms of the simulation, consistent with experimental data (see Figure 4 in [3]). **E)** The correlation of conditional expectation (CorCE) between various time bins during decision formation. Hot color indicates high CorCE. Compare to Figure 4D in [3].

dominant motion of $f_{\mathrm{dd}} = 0.55\mathrm{Hz}$. As the underlying rate of each neuron was accessible in the computer simulations, we were able to directly measure this variance. Figure 1C shows that the varCE of a given neuron in the circuit rises approximately linearly over the first 400ms of stimulus presentation. This is consistent with the experimental data, compare to Figure 4 in [3]. The model however shows a slight tendency for saturation, since the probability of a decision variable saturates at a value of 1.

The correlation structure of the conditional expectation was also studied. The authors state that, if neurons integrate information stochastically in a diffusion-like process, then spike counts at neighboring time bins during a single trial ought to covary. This is because such a mechanism implies that the spike rate at a given time is determined by the spike rate at the previous time bin plus a random increment [3]. Since more and more variance is added through time, the covariance should decrease as time bins are further separated in time. We determined the correlation of conditional expectation by computing the correlation coefficients

of the underlying rates between different time bins (50ms bin sizes). The result, illustrated in Figure 1D, is consistent with experimental data, compare to Figure 4D in [3]. More specifically, the two characteristic features of the experimental data were reproduced by our model: First, for any given time separation (matrix elements along the same juxtadiagonal), the CorCE increases as a function of time. Second, at any time (matrix elements along the same row), the CorCe is strongest in neighboring time bins and becomes weaker at larger temporal separation.

Methods

Here, we provide details on the computer simulations for the random-dot motion task. The particle filter circuit consisted of 1000 neurons per state and an estimation sample size of 200. The ensemble size of the action readout layer was 100 neurons. Considering eq. (4), we identified the random variable $v^Z(t)$ with the random variable estimated in the particle filter circuit (dominant dot-movement direction d). The random variable $v^X(t)$ has 2 states encoding reward for saccades to the two targets. The mapping between these random variables is straight forward with conditional probabilities given by $P(v^X = d|v^Z = d') = 1$ for $d = d'$ and $P(v^X = d|v^Z = d') = 0$ otherwise. With these conditionals, we obtained the synaptic weights through eq. (6) with $\alpha = 1$.

In all simulations, weights from evidence neurons to neurons in the evidence layer were set to values according to $f_{dd} = 0.55$Hz, also in Figure 1B where the evidence was generated with various f_{dd}'s. The lateral inhibition scaling was set to $I_0^{\text{lat}} = 5$.

The varCE was computed as follows. The dominant direction was kept fixed in all experiments with $f_{dd} = 0.75$Hz. One arbitrary neuron $z_{\text{ev},i}^n$ in layer $\mathcal{L}_{\text{dyn}}$ from the state that coded for the dominant movement direction was chosen. The expected spike count in a $\Delta T = 50$ms time bin was computed for each trial as $Count(t) = \int_{t-\Delta t}^{t} \rho_i^n(s)ds$. The varCE was given by the variance of $Count(t)$ over 500 trials. The $CorCE(t,t')$ was computed as the correlation coefficient between $Count(t)$ and $Count(t')$.

References

1. Gold JI, Shadlen MN (2007) The neural basis of decision making. Annu Rev Neurosci 30: 535–574.

2. Shadlen MN, Gold JI (2005) The neurophysiology of decision-making as a window on cognition. In: The cognitive neurosciences, MIT Press. 3rd edition, pp. 1229–1241.

3. Churchland AK, Kiani R, Chaudhuri R, Wang XJ, Pouget A, et al. (2011) Variance as a signature of neural computations during decision making. Neuron 69: 818–831.
