# Supplementary material for: Ensembles of Spiking Neurons with Noise Support Optimal Probabilistic Inference in a Dynamically Changing Environment
Source: PLoS Comput Biol. 2014 Oct 23;10(10):e1003859. doi: 10.1371/journal.pcbi.1003859 (PMC4207607; doi:10.1371/journal.pcbi.1003859)
Supplement: Text S5 — Probabilistic reasoning in area LIP. (PDF) [file pcbi.1003859.s006.pdf]

Supporting Text S5 for: Ensembles of spiking neurons with noise support optimal probabilistic inference in a dynamically changing environment

Robert Legenstein*, Wolfgang Maass,
Institute for Theoretical Computer Science
Graz University of Technology
A-8010 Graz, Austria
* **E-mail:** `robert.legenstein@igi.tugraz.at`

Probabilistic reasoning in area LIP

Yang and Shadlen [1] showed that the firing rate of neurons in parietal cortex of monkeys is proportional to the momentary log-likelihood ratio of a rewarded action for the given sensory evidence. Four symbols $s_1, \ldots, s_4$ were successively presented to the monkey, where each of them was independently drawn from a set of ten possible shapes, i.e, $s_i \in \{1, \ldots, 10\}$. Based on this information, the monkey had to decide whether to make an eye movement to a red or a green target to obtain reward. A weight w_i was assigned to each shape i and kept fixed during the whole experiment. The sum of the four weights associated with the shapes shown in a trial established the probability that reward would accompany one of the two choices [1].

Denoting the time of the presentation of the n-th shape as epoch n, the log-likelihood ratio (measured in units of bans) for an eye movement to the red target for symbols up to epoch n is defined as

$$\mathrm{logLR}_n = \log_{10} \frac{P(s_1, \ldots, s_n | \text{reward at red target})}{P(s_1, \ldots, s_n | \text{reward at green target})}. \quad (1)$$

For a red target, this value is exactly the sum of weights once all four shapes are shown ($n = 4$), and it is roughly proportional to the sum of the n shapes in the n-th epoch for $n < 4$. Neurons in LIP were shown to have firing rates proportional to the logLR.

We modeled this task by assuming that $P(s_1, \ldots, s_4 | \text{reward at red target})$ factorizes such that $P(s_1, \ldots, s_4 | \text{reward at red target}) = \prod_{i=1}^{4} P(s_i | \text{reward at red target})$. As we will see below, this is a very good approximation to the true likelihood. With this assumption, the task falls into task class B (evidence integration). The optimal solution can be approximated by particle filtering for a random variable with two hidden states (rewarded eye movement to the red or

green target respectively), see Figure 1A. Each shape was represented by one afferent neuron. In addition, we introduced afferent neurons that represented the presence of saccade-targets for reasons that are detailed below. See *Methods* in this supporting text for details on this simulation.

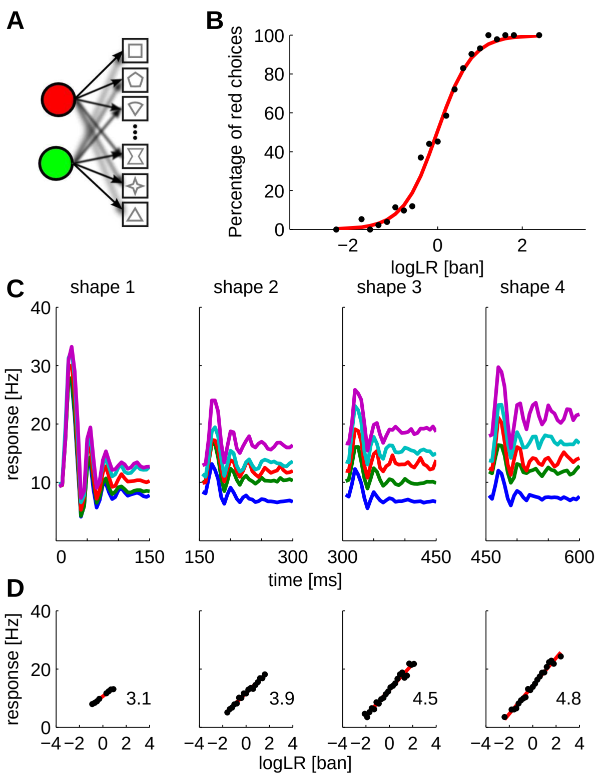

Figure 1. (Legend on next page)

The behavior of the model is illustrated in Figure 1B-D. It is similar to the experimental data in several respects. First, the percentage of a given choice increases with the logLR in favor of this decision according to a typical sigmoidal psychometric curve (Figure 1B). Second, the firing rate of network neurons is proportional to the logLR in any of the four epochs (Figure 1C, D) as observed in experiments. Note that the logLR on the x-axis of panel D is the true logLR. In other words, its calculation was exact, not based on the naive Bayes assumption used in the model. Finally, we see a typical activity transient due to strong network input when

Figure 1. Probabilistic reasoning through particle filtering. A) Estimated random variable. It has two states, encoding rewarded saccades to the red and green locations respectively. Each state gives rise to observed symbols with varying probability (indicated by blurriness of arrows). See Figure 5B in *Results* for the circuit architecture. **B)** Percentage of red choices as a function of the logLR for red (the choice was defined as the action with the highest probability at the end of epoch 4). Compare to Figure 1b of [1]. Red curve: sigmoidal fit to the data points (1000 trials in total). **C)** Effect of logLR on the ensemble rate of network neurons. Shown is the average firing rate in one ensemble during each epoch. The averages were computed for five quantiles of the logLR (i.e., in each epoch, the logLRs were divided into five sets of approximately equal size; all trials with a logLR in one of these sets in the epoch were averaged). Quantiles are indicated by color (from lowest to highest logLR: blue, green, red, cyan, magenta). Compare to Figure 3a in [1]. **D)** Firing rate is linearly related to the logLR. Mean firing rate was calculated for one network neuron over the last 75ms of each of the presentation epochs. The red line shows the least squares fit to the data points. Its slope is indicated on the bottom right of each panel. Compare to Figure 2c in [1]. In panels B and D, firing rates for identical logLRs were averaged to obtain the data points.

shapes appear (Figure 1C). This transient is caused by network input which leads to an increase of circuit activity that is compensated subsequently by lateral inhibition. The first transient is larger due to additional input from afferents that represent saccade targets. Hence, the strong response in the first epoch seen in experiments is explained in our model by transient responses to additional circuit input.

Methods

We simulated a particle filter circuit with ensemble size $M = 1000$ and estimation sample size $L = 400$; The lateral inhibition scaling was set to $I_0^{\mathrm{lat}} = 0.25$. No action readout layer was added.

12 evidence neurons provided input to the circuit. Two of them represented the saccade targets. Each of the remaining eight neurons represented one of the eight shapes that determined

the probability of reward. Spikes of the afferent neurons were produced as follows. First, the sequence of shapes $s_1, \ldots, s_4$ that was presented was drawn randomly. All possible sequences were equally probable. As in the experiment by Yang and Shadlen [1], the two 'trump' symbols were not presented. After 100ms waiting time, the two targets for the eye-movement and the first shape appeared. This caused a spike in the afferent neurons for the two targets and the afferent neuron for s_1 at a time that was uniformly distributed in $[100, 110]$ms. Subsequently, the afferent neuron for s_1 continued to spike in a Poissonian manner with rate $f_{\text{in}} = 10$Hz. The other three targets appeared at respective times 250ms, 400ms, 550ms and spikes in the corresponding afferent neurons were produced analogously.

To determine weights for connections from evidence neurons to neurons in the evidence layer, we used $\lambda_{jl} = P(s_i = j|a = l)10$Hz. Here, $P(s_i = j|a = l)$ is the probability that shape j is observed in epoch i, given that the rewarded action is l. Note that this probability is the same for all epochs i. It can be computed as follows:

$$P(s_1 = j|a = l) = \frac{P(s_1 = j, a = l)}{P(a = l)} = 2P(s_1 = j, a = l).$$

We obtain the joint via marginalization:

$$P(s_1 = j, a = l) = \sum_{\mathbf{s}:s_1=j} P(a = l|s_1, s_2, s_3, s_4)P(s_1, s_2, s_3, s_4) \propto \sum_{\mathbf{s}:s_1=j} P(a = l|s_1, s_2, s_3, s_4),$$

where the sum runs over all shape sequences where the first shape is j. The conditional for movement to the red target $P(a = R|s_1, s_2, s_3, s_4)$ is given by

$$P(a = R|s_1, s_2, s_3, s_4) = \frac{10^{\sum_i w_i}}{1 + 10^{\sum_i w_i}},$$

and the conditional for the green target is $P(a = G|s_1, s_2, s_3, s_4) = 1 - P(a = R|s_1, s_2, s_3, s_4)$ [1]. Here, w_i is the weight associated with symbol s_i that was set to $\mathbf{w} = (-70, -0.9, -0.7, -0.5, -0.3, 0.3, 0.5, 0.7, 0.9, 70)$. For the afferent neurons indicating the targets for saccades, λ_{ji} was set to 0.8Hz for both states i.

References

1. Yang T, Shadlen MN (2007) Probabilistic reasoning by neurons. Nature 447: 1075–1080.
